# Exhausted natural killer cells in adult IgA vasculitis

**DOI:** 10.1186/s13075-025-03559-y

**Published:** 2025-04-23

**Authors:** Matija Bajželj, Emanuela Senjor, Nika Boštic, Matjaž Hladnik, Snežna Sodin-Šemrl, Milica Perišić Nanut, Janko Kos, Alojz Ihan, Alojzija Hočevar, Andreja Nataša Kopitar, Katja Lakota

**Affiliations:** 1 Department of Rheumatology, University Medical Centre Ljubljana, Ljubljana, Slovenia; 2 Faculty of Mathematics, Natural Sciences and Information Technologies, University of Primorska, Koper, Slovenia; 3 Faculty of Medicine, University of Ljubljana, Ljubljana, Slovenia; 4 Department of Biotechnology, Jožef Stefan Institute, Ljubljana, Slovenia; 5 Faculty of Pharmacy, University of Ljubljana, Ljubljana, Slovenia

**Keywords:** IgA vasculitis, Adults, RNA sequencing, Nephritis, Natural killer cells

## Abstract

**Introduction:**

IgA vasculitis nephritis (IgAVN) manifests in up to 84% of adult patients with IgA vasculitis (IgAV) and is associated with an elevated risk of progression to chronic kidney failure. The underlying pathogenic mechanism of adult IgAVN in leukocytes remain largely uncharacterised. Although natural killer (NK) cells were investigated in paediatric IgAV, their specific role in the pathogenesis of adult IgAV has yet to be elucidated.

**Methods:**

RNA sequencing of leukocytes from adult IgAV patients and healthy controls (HC) was performed. NK cells’ cytotoxicity was assessed using calcein-AM stained K562 cells, and exocytosis was measured by LAMP-1/CD107a expression. Intracellular perforin and granzyme B were analyzed via flow cytometry, and cytokine secretion was measured by Luminex xMAP. Interferon-induced genes were validated with qPCR.

**Results:**

Principal component analysis (PCA) of leukocyte gene expression profiles distinguished IgAV patients from HC. Pathway enrichment analysis showed differences in patients’ subsets - Interferon signalling Reactome pathway was observed only in sample from patients with skin-limited IgAV (sl-IgAV) and was confirmed by increased expression of interferon-induced genes using qPCR. Only in samples from IgAVN patients enrichment of NK cell-mediated cytotoxicity KEGG pathway was found. NK cells from IgAVN patients showed significantly decreased cytotoxicity compared to samples from sl-IgAV patients (*p* = 2.53 × 10^− 2^). The % of CD107a^+^-NK cells significantly increased after stimulation in HC (*p* = 9.7 × 10^− 3^) and in sl-IgAV patient samples (*p* = 2.21 × 10^− 2^) while only a minor increase was observed in samples of IgAVN patients. IgAVN patients exhibited a decreased % of perforin^+^ NK cells compared to HC. Following phytohemagglutinin (PHA)/interleukin (IL)-2 stimulation, a significant reduction in intracellular perforin level was observed in HC (*p* = 2.53 × 10^− 2^), but not in IgAVN patients NK cells. Interferon (IFN)-ϒ and macrophage inflammatory protein (MIP)-1β were significantly decreased in NK cell culture supernatants from IgAVN patients (*p* = 2.64 × 10^− 2^ and *p* = 2.65 × 10^− 2^ respectively).

**Conclusion:**

Patients with IgAVN exhibited impaired cytotoxic and immunomodulatory functions of NK cells, along with a marked absence of interferon signaling in PBMCs. Further studies are needed to confirm if discrimination of patient subsets based on leukocyte samples might be of clinical use and if deregulated NK function might contribute to the pathogenesis of nephritis in adult IgAV.

**Supplementary Information:**

The online version contains supplementary material available at 10.1186/s13075-025-03559-y.

## Introduction

Immunoglobulin A vasculitis (IgAV) is a form of small vessel vasculitis characterised by a heterogeneous clinical presentation [1]. In adults, IgAV can manifest as a potentially life-threatening condition, in contrast to its typically self-limiting course in pediatric cases [2]. The incidence of IgAV in adults is underestimated, as shown by our previous study [3]. While cutaneous vasculitis, presenting as palpable purpura and/or petechiae, is a hallmark feature, systemic involvement is frequently observed, including renal, articular, and gastrointestinal (GIT) manifestations [4]. Renal involvement occurs in up to 84% of adult patients and is associated with an increased risk of progression to chronic kidney disease [5]. Although glucocorticoids are the mainstay of IgAV treatment, primarily alleviating GIT symptoms and joint pain, their efficacy in preventing the progression to chronic kidney disease appears to be limited [6].

While therapeutic guidelines in adult IgAV have not been established yet, in cases presenting with organ- or life-threatening complications, such as severe renal involvement, a combination of glucocorticoids and immunosuppressive agents is routinely used [7]. However, the efficacy of these treatment regimens remains a subject of debate. The majority of available studies were conducted in paediatric populations, with findings often extrapolated to adult patients [8]. Furthermore, and to our knowledge, the only randomised study performed on adults with IgAV shows that the addition of cyclophosphamide to corticosteroids in patients with renal involvement is not of substantial benefit [9]. Additionally, IgAV may recur following kidney transplantation, which often leads to graft loss [10, 11].

Currently, the pathogenic mechanism of adult IgAV at the molecular and cellular levels has been poorly explored, leading to a difficult selection of potential cellular targets and biomarkers for optimized therapeutic strategies. Research investigating the role of innate immune cells in IgAV has predominantly focused on neutrophils, revealing altered neutrophil counts in patients’ peripheral blood [12] and their presence within inflammatory infiltrates in the skin [13]. However, there is a paucity of studies examining the functional role of other immune cell populations, such as NK cells, in the pathogenesis of adult IgAV. NK cells, classified among cells of the innate immune response, play a dual role by directly eliminating tumour cells and pathogen-infected cells while also modulating immune responses through cytokine secretion [14]. Full activation of NK cells requires stimulation by type I interferons or proinflammatory cytokines [15]. NK cells are categorized into two major subsets based on their surface expression of CD56 and CD16. CD56^dim^/CD16^+^ cells are predominant in peripheral blood, exerting cytotoxic activity, while CD56^bright^/CD16^dim^ are considered to have weak cytotoxic activity, but prominent immunoregulatory properties with abundant cytokine production [16]. Functionally, NK cells mediate cytotoxicity by degranulating vesicles containing cytotoxic molecules, perforins and granzymes, upon recognition of susceptible target cells. This process can be assessed by measuring the surface presence of vesicle-associated protein CD107a using flow cytometry, which reflects cytotoxic activity [17]. Besides cytotoxic function NK cells exert immunomodulatory effects through the secretion of key cytokines, such as interferon (IFN)-ϒ, tumor necrosis factor (TNF)-α, macrophage inflammatory protein (MIP)-1α, and MIP-1β [18]. While studies in pediatric IgAV and IgA nephropathy reported a reduction in both the number and function of NK cells [19, 20], their involvement in the pathogenesis of adult IgAV remains to be elucidated.

Our first aim was to identify dysregulated biological processes in leukocytes from adult IgAV patients using bulk RNA sequencing. RNA-Seq analysis indicated enrichment in deregulated genes involved in NK cell cytotoxic pathway in adult IgAV patients with renal involvement. Based on these findings, we further investigated the specific role of NK cells in the pathophysiology of IgAV by assessing their frequency, phenotype, cytotoxic capacity, and immunomodulatory function. The observed impairment in NK cell activity may serve as a potential biomarker for renal involvement in adult IgAV.

## Methods

### Study design, study subjects and clinical data collection

Adults (aged ≥ 18) with histologically proven IgAV, diagnosed for the first time between July 1, 2020 and September 30, 2023, participated in the unicenter study at the University Medical Centre Ljubljana (UMCL). Patients’ data were collected in a prospective manner. All patients underwent an extensive clinical evaluation and laboratory workup. All subjects signed informed consent. The study was approved by the Slovene National Medical Ethics Committee (#159/07/13, #99/04/15, #65/01/17 and #0120–121/2021/3).

In the study the definitions of extensive purpura (i.e. purpura above waistline), GIT and renal involvement were used as previously reported in detail [21]. The diagnosis of IgAV was established according to the 2012 revised International Chapel Hill Consensus Conference Nomenclature of Vasculitides [1]. All included IgAV patients fulfilled the 2010 European League Against Rheumatism/Paediatric Rheumatology International Trials Organisation/Paediatric Rheumatology European Society (EULAR/PRINTO/PRES) classification criteria [22].

Venous blood was collected at the time of initial diagnosis, before treatment initiation. IgAV-related signs and symptoms data were collected as well as age, sex, body mass index (BMI), duration of clinical symptoms, past and concurrent infections. Disease activity was determined using Birmingham vasculitis activity score (BVAS) [23]. Healthy controls (HC) were investigated at the Department of Rheumatology, UMCL for the presence of systemic autoimmune rheumatic diseases (SARD) and immunosuppressive treatment, as exclusion criteria.

### Laboratory parameters

C-reactive protein (CRP), serum amyloid A (SAA), complete differential blood count, basic biochemistry panels including electrolytes, creatinine, urea, serum protein electrophoresis, serum IgA, M, G levels, complement components C3 and C4 and urine analysis were determined for routine diagnostics.

### RNA extraction, sequencing and bioinformatic analysis

Venous blood was collected in 4 ml EDTA tubes and filtered using the LeukoLock fractionation and stabilization kit (Invitrogen) according to manufacturer instructions. Leukocytes were lysed and the lysate was stored at -80^o^C until the RNA isolation process. RNA was isolated using binding beads (LeukoLock fractionation and stabilization kit (Invitrogen)) according to the manufacturer protocol. The quantity and quality of the RNA obtained were checked using NanoDrop 2000 spectrophotometer (Thermo Fisher Scientific, Waltham, MA, USA).

The isolated RNA from leukocytes was used for RNA-Seq library preparation with TruSeq RNA Access Library Prep Kit (Gold, Ilumina, San Diego, CA, USA). Paired-end Sequencing, 2 × 100 bp, was performed using the Illumina HiSeq 4000 platform (25.9 to 30.8 million reads were obtained per sample). RNA-Seq data was analysed as described by Bajželj et al. [21]. After quality control and adapter removal with FastQC v0.11.9 and Trimmomatic v0.36, reads were mapped to reference transcriptome (Ensembl release 104) with Salmon v1.5.2 [24]. The salmon transcriptome index was prepared using the transcriptome (cDNA.all.fa and ncrna.fa) and genome reference files, with the latter used as a decoy sequence. Mapping was performed with the options validateMappings, seqBias, and gcBias. Transcript level quantification was summarized to the gene level using tximeta R package (v1.8.5). Genes with fewer than 10 counts across all samples were removed. Statistically significant differences in gene expression in leukocytes from IgAVN (*n* = 3), sl-IgAV (*n* = 3), and age-and sex-matched HC (*n* = 3) were determined using the Wald test and adjusted p-values were calculated with the Benjamini and Hochberg procedure implemented in DESeq2 package (v1.30.1) [25]. Additionally, log-fold change values were corrected with the apeglm method (approximate posterior estimation for generalized linear models) implemented in lfcShrink DESeq2 function [26]. Finally, differentially expressed genes were defined with corrected log2 fold change values ≥ │1│and p-adj < 0.05.

Distinct patterns of gene expression between patient subgroups and HC were examined using a principal component analysis (PCA) with the plot PCA function, considering all genes. Count data transformed with the regularized logarithm (via the DESeq2 rlog function)) was used for PCA analysis. Over representation analysis (ORA) of Kyoto Encyclopedia of Genes and Genomes (KEGG) and Reactome pathways was performed with R package clusterProfiler [27]. Hypergeometric test was employed to determine if the observed enrichment exceeded what might be expected by chance.

### Quantitative real-time polymerase chain reaction (qPCR)

After LeukoLock (Invitrogen) isolation of RNA from peripheral blood leukocytes, a total of 1 µg of cDNA was synthesized from isolated RNA using Reverse Transcription System (Promega, Madison, WI, USA) according to manufacturer’s instructions. qPCR was performed using 5× HOT FIREPol EvaGreen qPCR Mix Plus (Solis Biodyne, Tartu, Estonia) on the LightCycler 480 System (Roche). Primers were custom-made and purchased from IDT (Coralville, IA, USA) with the sequences indicated in Table S1, tested by melt curves and standard curves. Data were presented as a relative expression, normalized to GAPDH, and calculated by the 2−∆∆CT method. The endogenous control expression was tested to be stable regardless of the treatment groups.

### Isolation of peripheral blood mononuclear cells (PBMCs)

Blood was collected in two 6 ml EDTA tubes and processed under sterile conditions within 2 h. Peripheral blood was layered onto Ficoll-Paque and centrifuged at 400 g (brake-off) for 30 min at 25 °C. The interface containing mononuclear cells was collected and washed in phosphate-buffered saline (PBS). Pellets were suspended in freezing media and preserved in liquid nitrogen until analysis.

### Primary NK cell isolation

PBMCs were thawed and left to recover overnight in RPMI 1640 media supplemented with 8% heat-inactivated FBS, MEM non-essential amino acids (Gibco), 1 mM sodium pyruvate (Gibco), 1% P/S (pNK media). Subsequently, NK cells were isolated utilizing a negative selection magnetic isolation kit for NK cells (cat. 130-092-657, Miltenyi Biotec, Germany). The purity of isolated cells was assessed using flow cytometry (Attune NxT flow cytometer, Thermo Fisher Scientific, Massachusetts, USA) with anti-CD3 (cat. 130-113-128) and anti-CD56 (cat. 47-0567-42, Thermo Fisher Scientific) antibodies as presented in Figure S1. Flow Jo software (TreeStar, Oregon, USA) was used for analysis.

### Calcein-AM release assay

Isolated primary NK cells were activated overnight with 1000 IU/mL IL-2 (cat. 4030758, Bachem, Switzerland) [28, 29], and prepared at selected effector-to-target ratios, the highest being 5:1, with 5 serial dilutions in 96-well U-bottom plates. K562 target cells (*n* = 5000/well) were stained with 15 µM calcein-AM (cat. 17783, Sigma) in serum-free media for 30 min, washed and added to effector cells. The plate was centrifuged at 200 g for 1 min and incubated for 4 h at 37 °C with 5% CO2. After incubation, the plate was centrifuged at 700 g for 5 min, and 50 µl of supernatant was transferred to a new microtiter plate for fluorescence measurements. The released calcein-AM was measured using the Tecan M1000 microplate reader at 496 nm excitation and 516 nm emission. Cytotoxicity percentage was calculated as: 100 × (test release − spontaneous release)/(total release − spontaneous release). Spontaneous release of calcein-AM was determined in wells containing only target cells, whereas total release measurement was achieved by the addition of 2% Triton X-100 to the media to achieve target cell lysis. Lytic units (LU) were calculated using the inverse of the number of effector cells needed to lyse 30% of the target cells × 100.

### Degranulation assay

0.5 × 10^6^ PBMCs/ml were stimulated with 1.25 µg/mL phytohaemagglutinin (PHA) and 0.05 µg /mL IL-2. Cells were incubated 48 h at 37 °C in a cell culture incubator with 5% CO2. Cytotoxic granules release, determined as exposed lysosomal membrane protein (LAMP-1) /CD107a, was measured by flow cytometry to determine the cytotoxic ability of NK cells. For intracellular granzyme B and perforin staining, 0.5 × 10^6^ PBMCs/ml were stimulated with 50 ng/ml phorbol 12-myristate 13-acetate (PMA) (Sigma, Poole, UK) and 1 µg/ml ionomycin (Sigma) for 5 h at 37 °C.

### Flow cytometry

For CD107a measurement, PBMCs were washed with 2 ml of staining buffer (PBS with 1% bovine serum albumin (BSA) and stained with a cocktail of monoclonal antibodies: CD3 FITC, CD16 PerCP, CD56 APC, CD107a PE, all from BD Bioscience, for 20 min. Erythrocytes were lysed with BD FACS Lysing Solution (BD Bioscience) and washed twice with staining buffer. For the measurement of granzyme B and perforin, the cells were fixed with Cytofix buffer (eBioscience) for 15 min and then washed with 2 ml staining buffer. Cells were stained for 20 min for cell surface markers CD16 and CD56, washed with staining buffer, and then permeabilized with BD Perm/Staining buffer for 15 min. Cells were stained with antibodies against granzyme B (V450) and perforin (AlexaFluor 647) (BD Bioscience) diluted in BD Perm/Staining buffer. All incubations were performed in the dark at room temperature. The lymphocyte population was identified by cell size and granularity using light-scattering properties [forward scatter (FSC) versus side scatter (SSC)]. The percentage of NK cells was expressed as a proportion of total gated lymphocytes. NK cells were defined as CD56^+^ and/or CD16^+^ lymphocytes and categorized into 6 different subsets according to the intensity of CD56 and CD16 expression, and expressed as a proportion of total gated NK cells. Gates were set using appropriate negative controls for each intra- and extracellular antibody. For the gating strategy see Supplementary Figure S1. Representative overlay histograms for exposure of CD107a for unstimulated and stimulated conditions for IgAVN patients and HC are provided in Figure S3. At least 30,000 cells per sample were collected using a BD Canto II flow cytometer. Data were analyzed using FlowJo 10.10 and BD FACSDiva v8.0.1 software (TreeStar Inc.) (BD Biosciences, San Jose, CA, USA).

### Luminex assay

Luminex assay was performed in supernatants from NK cells isolated from IgAV patients and age-/sex-matched HC activated overnight with 1000 IU/mL IL-2 (cat. 4030758, Bachem, Switzerland). Concentrations of 8 analytes including chemokine (C-C motif) ligand 3 (CCL3), chemokine (C-C motif) ligand 4 (also CCL4), granulocyte-macrophage colony-stimulating factor (GM-CSF), granzyme B, interferon gamma (IFN-γ), interleukin (IL) 8, IL-10 and TNF-α were measured by the Luminex platform (MAGPIX System, Merck, Darmstadt, Germany) using human pre-mixed multi-analyte kits (R&D Systems), according to manufacturer instructions.

### Statistical analysis

Data were analyzed with GraphPad Prism version 8.0. The normality of data distribution was investigated by the Shapiro-Wilk and Kolmogorov-Smirnov normality tests. Normally distributed data were presented as mean; for two-group comparison 2-tailed parametric *t-test* was used and when more than 2 groups were compared, a 1-way analysis of variance (ANOVA) test was performed. For non-normally distributed data median and interquartile range is presented, the Mann-Whitney test was used for two-group comparison and the Kruskal-Wallis test was used for multiple-group comparison. To adjust for multiple comparisons Tukey’s (variances not equal) or Dunn’s *post hoc* test were used. A p-value of < 0.05 was considered statistically significant, n refers to the number of biological replicates.

## Results

### Comparison of the leukocyte transcriptomic profiles from all IgAV patients, IgAVN, sl-IgAV and HC

Six adult treatment-naïve IgAV patients were consecutively recruited at the time of diagnosis for RNA sequencing of leukocytes. Among them, three had skin-limited IgAV (sl-IgAV), while three had IgAVN. The HC group consisted of three age-matched females (Table 1).

**Fig. 1:**
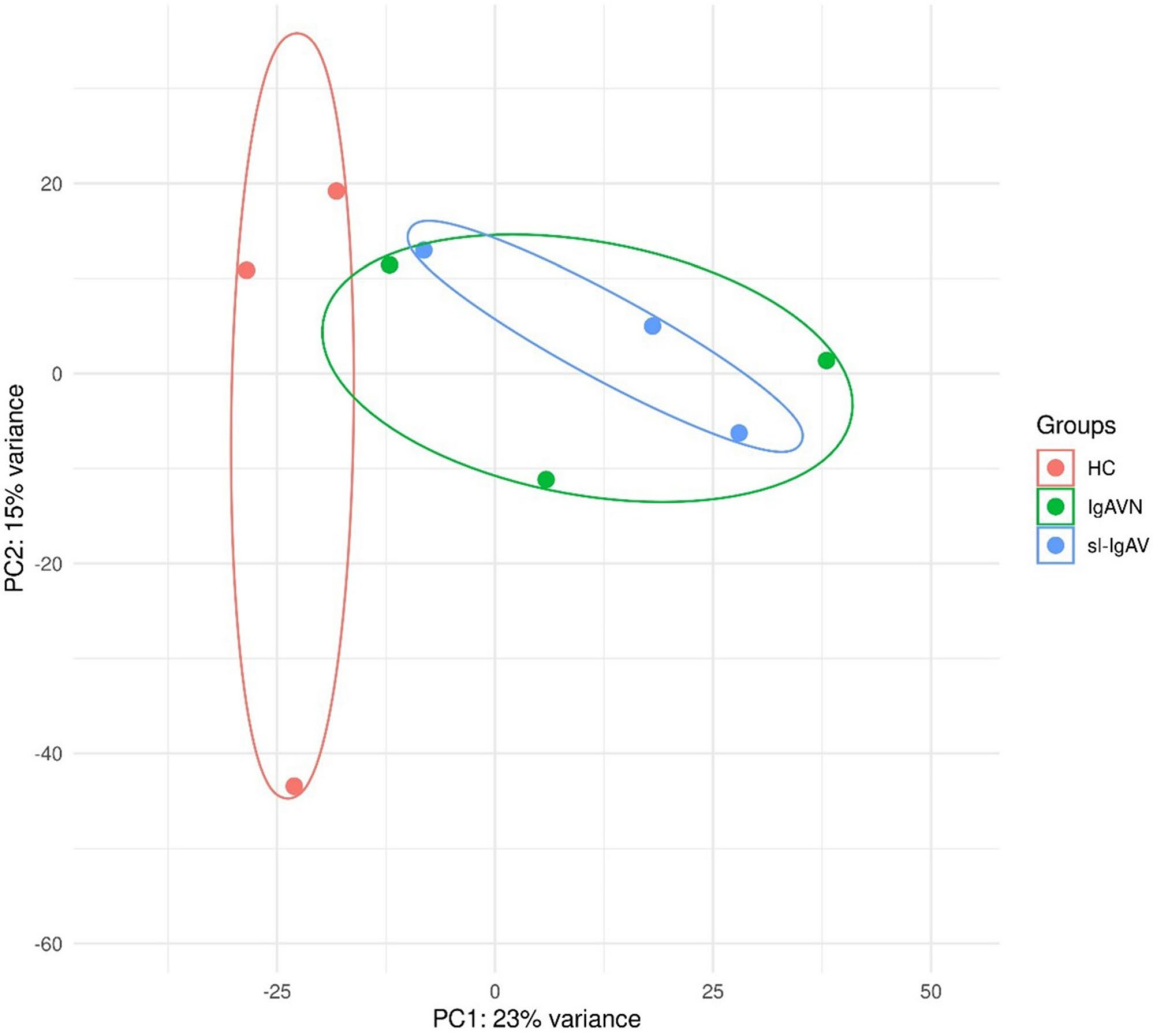
PCA distinguished IgAV patients from HC. Overlapping gene expression was observed between IgAVN and sl-IgAV patients. PCA was performed with the gene expression count data, previously transformed with the regularized logarithm (as implemented in the DESeq2 rlog function). Samples that cluster closer together on the plot share more similar gene expression patterns. PCA, principal component analysis; IgAV, immunoglobulin A vasculitis; HC, healthy controls; IgAVN, IgA with renal involvement; sl-IgAV, skin-limited IgAV

Principal component analysis (PCA) revealed that all IgAV patients can be distinguished (Fig. 1) from HC, while overlapping gene expression was observed between IgAVN and sl-IgAV. Differential expression analysis identified 174 differentially expressed genes (DEGs) in all IgAV patients compared to HC, with the majority of genes being upregulated. Patients with IgAVN exhibited 234 DEGs, whereas those with sl-IgAV had 121 DEGs compared to HC. A total of 44 common DEGs were shared between IgAVN and sl-IgAV, suggesting the presence of specific gene patterns for individual patient groups (Table 2).

### Analysis of deregulated biological processes in IgAV leukocytes– pathway enrichment analysis

**Fig. 2:**
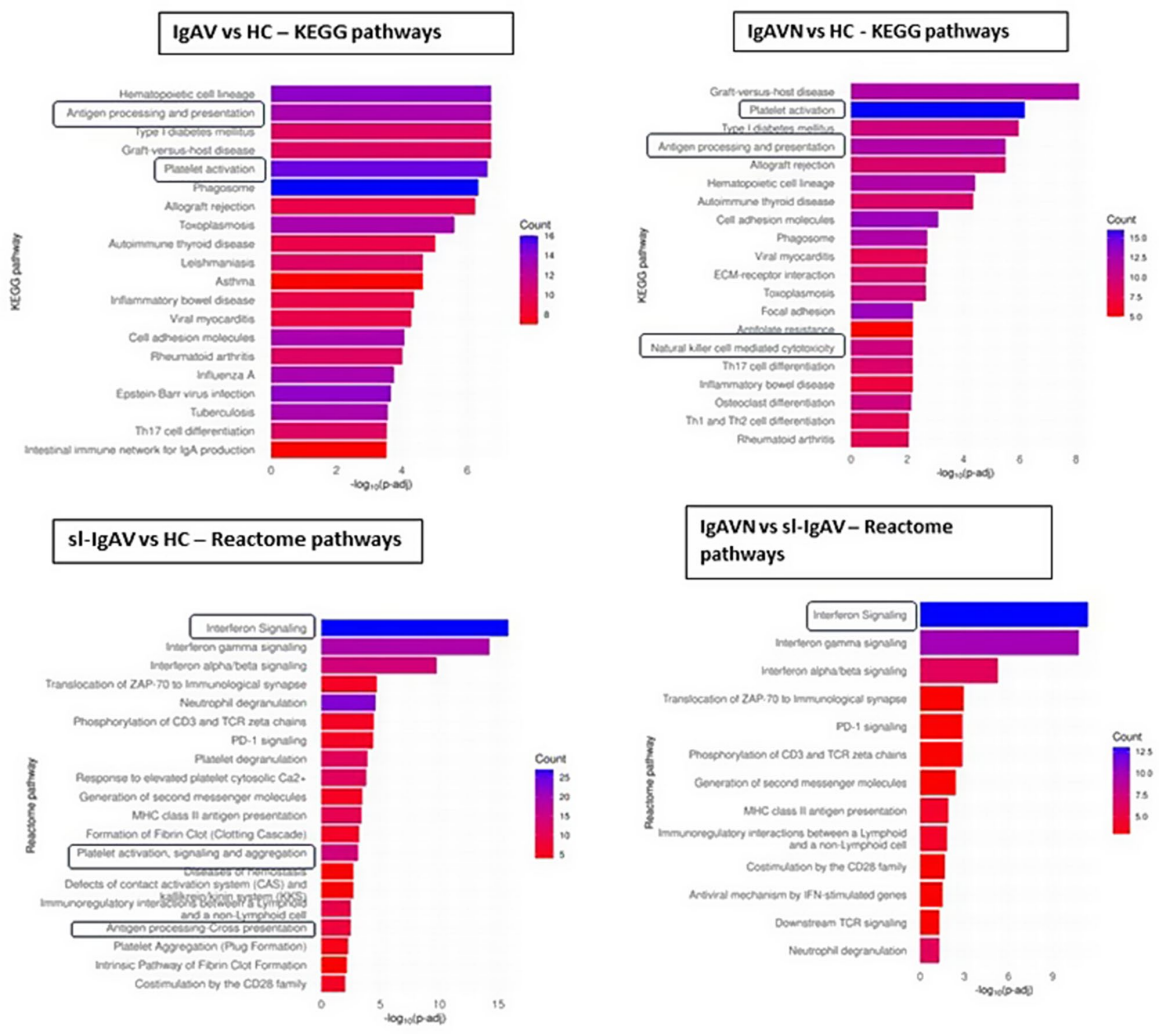
ORA of enriched KEGG or Reactome pathways. Each bar on the graph represents a biological pathway, such as KEGG or Reactome pathway. Enriched terms are presented with color-coded columns, blue colors indicate higher number of genes associated with respective pathway and column length indicate significance. ORA, over-representation analysis; KEGG, Kyoto Encyclopedia of Genes and Genomes; IgAV, immunoglobulin A vasculitis; HC, healthy controls; IgAVN, IgAV with renal involvement; sl-IgAV, skin-limited IgAV; p-adj, adjusted p-value

ORA of KEGG Pathways revealed Antigen processing and presentation (p-adj = 1.84 × 10^− 7^) and Platelet activation (p-adj = 2.37 × 10^− 7^) among most enriched pathways in all IgAV compared with HC. Specifically, in the IgAVN group compared to HC, ORA revealed the enrichment of NK cell-mediated cytotoxicity (p-adj = 7.06 × 10^− 3^) KEGG pathway. On the other hand, sl-IgAV versus HC exhibited enriched NOD-like receptor signaling KEGG pathway (p-adj = 2.26 × 10^− 5^) and Interferon signaling Reactome pathway (p-adj = 1.38 × 10^− 16^), suggesting the involvement of different immune cells and signaling pathways between IgAVN and sl-IgAV. Also, comparison IgAVN versus sl-IgAV ORA revealed the enrichment of both type I (p-adj = 5.25 × 10^− 6^) and type II Interferon Signalling Pathway (p-adj = 1.63 × 10^− 11^) (Fig. 2) with the majority of genes from these pathways being upregulated in sl-IgAV. Enrichment analysis of Reactome and KEGG pathways suggested the involvement of NK cells and the absence of interferon signature in IgAVN patients.

### NK cells from patients with IgAVN showed reduced ability to kill target cells

Transcriptomic analysis revealed the downregulation of prototypic NK cell marker CD56, activating NK cell receptors (KLRC1, KLRC2, KLRD1), as well as effector enzymes (granzyme B, M and H, perforin 1) mediating cytotoxic functions in IgAVN patients (Table S1). These findings suggest a reduction in NK cell activity or number in adult IgAVN patients. The % of NK cells was not significantly changed between IgAVN patients, sl-IgAV and HC (Figure S4). Therefore, we further investigated whether NK cell cytotoxic function is impaired in IgAVN patients.

Sixteen treatment-naïve adult IgAV patients (9 males, 7 females, median (IQR) age 68.7 (52.6–81.9 years) were recruited for collection of PBMCs and NK cell isolation. The HC group consisted of 12 age-matched individuals (Table 3).

**Fig. 3:**
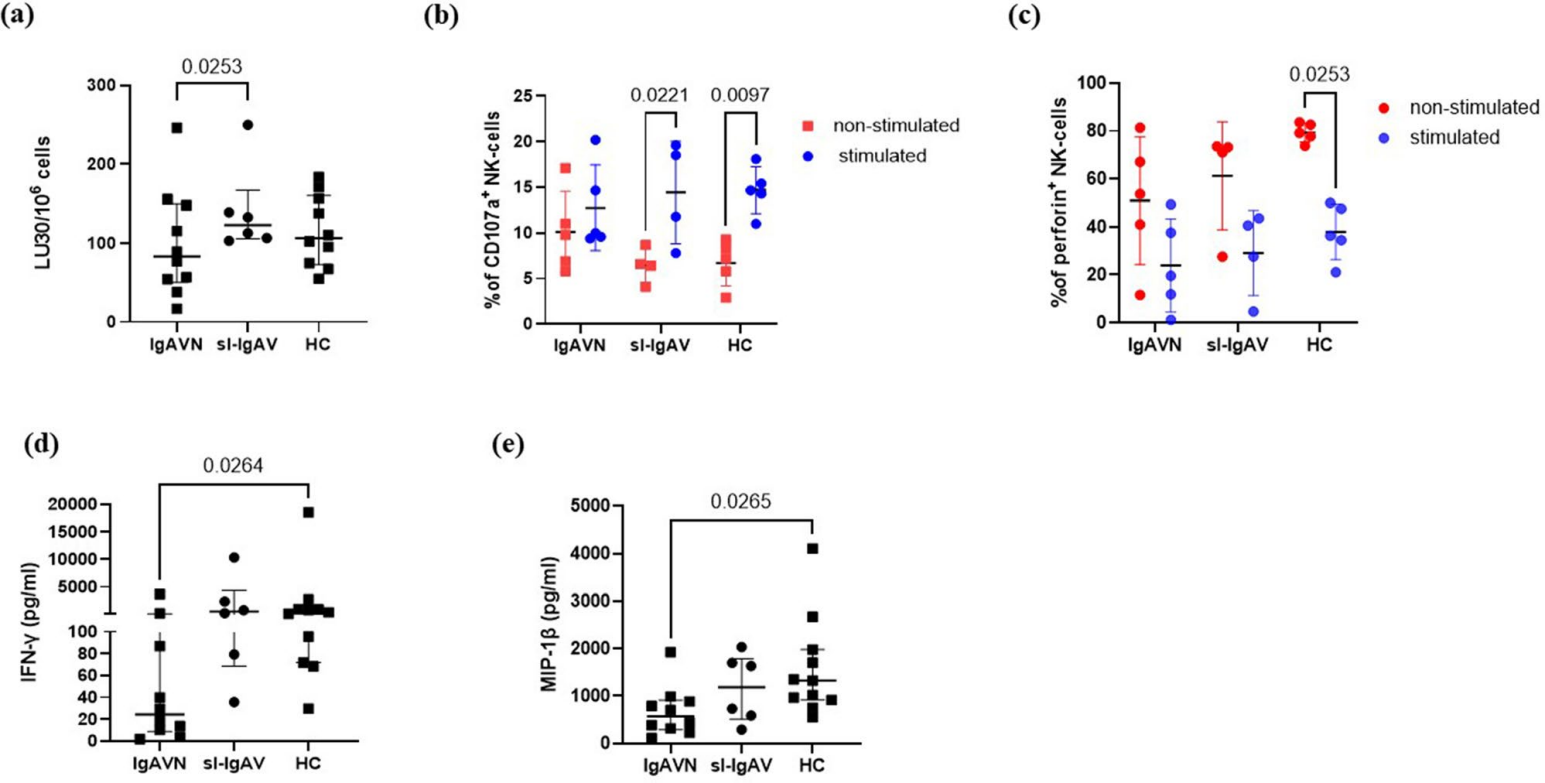
Disturbed NK cell function in IgAVN patients. **(a)** Decreased cytotoxicity of NK cells in IgAVN compared to sl-IgAV patients. NK cells from HC, IgAVN and sl-IgAV were incubated with calcein-AM-labelled K562 cells. LU 30/10^6^ cells were calculated using the inverse of the number of effectors needed to lyse 30% of the tumour cells × 100. **(b)** The number of CD107a^+^ NK cells significantly increased after stimulation with PHA and IL-2 in HC and in sl-IgAV patients while only a minor increase was observed in IgAVN patients. **(c)** After 5 h of incubation with PMA and ionomycin there was a limited decrease in intracellular perforin in IgAVN patients, while decrease was significant in HC. IFN-γ **(d)** and MIP-1β **(e)** were significantly decreased in IgAVN patients’ cell culture supernatants after stimulation with IL-2 compared to HC. Data are expressed as medians (Q25-Q75) of each group. NK cells, natural killer cells; IgAV, immunoglobulin A vasculitis; HC, healthy controls; IgAVN, IgAV with renal involvement; sl-IgAV, skin-limited IgAV; LU, lytic units; PHA, phytohaemagglutinin; IL-2, interleukin-2; PMA, phorbol 12-myristate 13-acetate; IFN-γ, interferon-γ; MIP-1β, macrophage inflammatory protein-1β

Isolated NK cells were activated overnight with IL-2, and their cytotoxicity was assessed using the calcein-AM release cytotoxicity assay. The purity of isolated NK cells in % of CD3^−^, CD56^+^ cells is provided in Table S3. A representative graph showing the percentage of cytotoxicity for each effector-to-target (E: T) ratio is presented in Figure S5. NK cells isolated from IgAVN patients showed significantly decreased cytotoxicity to calcein-AM stained K562 cells compared to sl-IgAV (*p* = 2.53 × 10^− 2^) (Fig. 3a).

### Degranulation is diminished in NK cells from IgAVN patients

To further elucidate the mechanism underlying the impaired NK cell cytotoxic function in adult IgAV, cytotoxic granule release from NK cells was assessed by measuring exposed LAMP-1/CD107a, a marker for degranulation. PBMC were only available from 5 IgAVN patients, 4 sl-IgAV patients and 5 HC to perform this assay. PBMC were stimulated with PHA and IL-2, incubated for 48 h and CD107a expression was measured by flow cytometry. Six distinct NK cell subpopulations were defined including CD56^bright^CD16^−^, CD56^bright^CD16^dim^, CD56^dim^CD16^−^, CD56^dim^CD16^dim^, CD56^dim^CD16^bright^, and CD56^−^CD16^bright^, as expected and previously described in the literature [30] (Figure S1). The % of NK cell subpopulations were not significantly changed between IgAVN patients, sl-IgAV and HC (Figure S4). The % CD56^dim^CD16^bright^ NK cells, the major cytotoxic subset in peripheral blood, was reduced in IgAVN patients compared to sl-IgAV and HC, however the difference was not statistically significant (median (IQR) of 19.8 (6.7–28.3) vs. 39.3 (9.65–57.4) and 33.7 (32.8–45.9) % of CD56^dim^CD16^bright^ NK cells)) (Figure S4). Unstimulated NK cells from IgAVN patients had more NK cells positive for CD107a^+^ compared to sl-IgAV and HC (median (IQR) of 9.8 (6.35–14.05) vs. 6.5 (4.675–8.15) and 7.2 (4.35–8.8) % of CD107a^+^ NK cells, respectively), although this difference did not reach statistical significance (*p* = 2.56 × 10^− 1^) (Figure S6a). Following PHA and IL-2 stimulation, the percentage of CD107a⁺ NK cells significantly increased in HC (*p* = 9.7 × 10^− 3^) and sl-IgAV patients (*p* = 2.21 × 10^− 2^), whereas no significant increase was observed in IgAVN patients (*p* = 6.35 × 10^− 1^) (Fig. 3b). The same was observed in major cytotoxic, CD56^dim^CD16^bright^ NK cell population (where CD107a⁺ population significantly increased in HC (*p* = 2.95 × 10^− 2^) and sl-IgAV (*p* = 1.90 × 10^− 2^), while no increase was observed in IgAVN patients (*p* = 7.87 × 10^− 1^)). Reduced capacity for cytotoxic vesicle degranulation in IgAVN patients NK cells suggests NK cell exhaustion in IgAVN patients, as their cytotoxic activity did not increase upon stimulation. NK cells from IgAVN patients exhibited significantly lower basal perforin expression compared to HC (*p* = 4.67 × 10^− 2^) (Figure S6b). Following 5 h of stimulation with PMA and ionomycin, a significant reduction in intracellular perforin levels was observed in HC (*p* = 2.53 × 10^− 2^), but not in IgAVN patients, further supporting a limited degranulation capacity in IgAVN (Fig. 3c). In contrast, granzyme B expression remained unchanged between IgAV patient subgroups and HC, both before and after stimulation (Figure S7).

### NK cells from IgAVN patients showed reduced IFN-γ and MIP-1β secretion in response to IL-2 stimulation

To investigate the immunomodulatory function of NK cells, we measured the secretion of IFN-γ, granzyme B, GM-CSF, MIP-1α and MIP-1β, TNF-α, IL-10 and IL-8 in cell culture supernatants following stimulation with IL-2 on samples used for cytotoxic calcein-AM assay using multiplex assays (Luminex xMAP technology). IFN‐γ (*p* = 2.64 × 10^− 2^), the most distinctive NK cell cytokine and MIP-1β (*p* = 2.65 × 10^− 2^) were significantly reduced in IgAVN patient supernatants after IL-2 stimulation compared to HC (Fig. 3d, e). while other cytokines were not significantly changed between IgAV patient groups and HC (data not shown).

### sl-IgAV patients exhibit an interferon signature

**Fig. 4:**
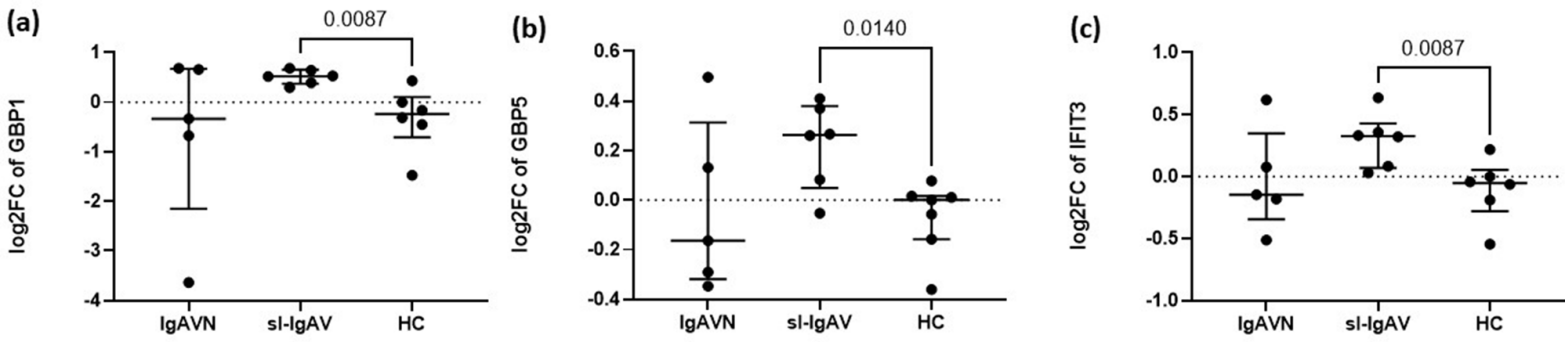
Expression of interferon-induced genes in IgAV patients’ leukocytes. Expression of GBP1 **(a)**, GBP5 **(b)** and IFIT3 **(c)** was significantly increased in sl-IgAV patients compared to HC. Data are expressed as medians (Q25-Q75) of each group. GBP1, guanylate binding protein 1; GBP5, guanylate binding protein 5; IFIT3, interferon-induced protein with tetratricopeptide repeats 3; IgAV, immunoglobulin A vasculitis; HC, healthy controls; IgAVN, IgAV with renal involvement; sl-IgAV, skin-limited IgAV

Overrepresentation analysis (ORA) identified enrichment of interferon signalling pathways in sl-IgAV patients, with upregulation of interferon-induced genes compared to both IgAVN patients and HC. To validate these findings, qPCR was performed, confirming the increased expression of three interferon-induced genes GBP1 (*p* = 8.7 × 10^− 3^), GBP5 (*p* = 1.4 × 10^− 2^) and IFIT3 (*p* = 8.7 × 10^− 3^) in 6 sl-IgAV patients compared to 6 HC, but not compared to 5 IgAVN patients (Fig. 4).

## Discussion

To our knowledge, this is the first study to report an IgAV-associated leukocyte transcriptomic signature in adults. In a non-hypothesis driven approach the deregulation of NK cell-mediated cytotoxicity at the transcriptomic level in patients with IgAVN was identified. Functional analyses confirmed a significant reduction in NK cell cytotoxic activity in IgAVN patients compared to those with sl-IgAV. Results also showed that a decreased NK cell cytotoxic function in IgAVN patients might be related to decreased levels of intracellular perforin. Notably, NK cells from IgAVN patients exhibited features of exhaustion, as they failed to enhance the degranulation of cytotoxic granules upon stimulation. NK cells isolated from IgAVN patients also demonstrated impaired immunomodulatory function. These findings provide novel insight into disturbed NK cell function in adult IgAV.

The leukocyte transcriptome could distinguish between IgAV patients and HC. Notably, two IgAV patients who were closest to the HC group in the two principal component spaces exhibited symptoms 2 and 110 days, respectively. This deviation from median symptom duration of other patinets might explain their gene expression resembling those of HC. Moreover, the partial overlap between IgAVN and sl-IgAV patients suggests the activation of shared pathogenetic mechanisms in both groups. This is supported by the enrichment of the Antigen processing and presentation and Platelet activation KEGG pathways in both patient groups. However, distinct DEGs identified between patient groups and HC suggests the involvement of additional pathogenic processes specific for each group, such as decreased NK cell function only in patients with IgAVN.

The role of NK cells in adult IgAV remains largely unexplored. In paediatric IgAV cases, a reduction in both NK cell numbers and cytotoxic activity were reported [19]. Interestingly, our current study, alongside with finding by Kuret et al. [12] demonstrates that percentage of NK cells in adult IgAV does not significantly differ from that in HC. The cytotoxic function of NK cells is regulated by delicate balance between activating and inhibitory receptors, as well as various environmental factors, including cytokines and NK cell receptor ligands [31]. Our RNA sequencing data revealed dysregulated expression of NK cell receptors in IgAVN patients. Furthermore, we observed elevated serum levels of two major NK cell stimulatory cytokines [32], IL-15 and IL-18, in cohort of 59 adult IgAV patients [33], indicating active stimulation for NK cell activation. We noted an increased baseline expression of CD107a on NK cells from IgAVN patients, combined with only a minimal increase in CD107a expression following stimulation in contrast to the responses observed in sl-IgAV and HC. This suggests that NK cells in IgAVN patients are already activated, but with more stimulation become exhausted, resulting in reduced cytotoxic activity. Furthermore, unstimulated NK cells from IgAVN patients demonstrated decreased intracellular perforin levels compared to HC, highlighting a deficiency in cytotoxic enzyme content. While our RNA sequencing revealed significantly reduced expression of granzyme B, intracellular levels of this protein did not show corresponding decline. Disrupted CD107a and perforin expression results in less granzyme B entering target cells, leading to reduced cytotoxic function [34]. Our transcriptomic analysis further identified decreased expression of *granzyme H* and *granzyme M* in IgAVN patients; these granzymes are predominantly expressed in NK cells [35, 36], suggesting that their decreased expression could also contribute to impaired NK cell cytotoxicity.

NK cells are the predominant source of IFN-γ among innate immune cells [37], although CD4^+^ Th1 cells and CD8^+^ cytotoxic T cells can also secrete this cytokine [38]. Our findings indicate a significant reduction in IFN-γ secretion by NK cells from IgAVN patients. However, previous studies that measured IFN-γ levels in plasma or serum did not report significant differences between IgAV patients and HC [39, 40].

Recent study has reported a reduced number of NK cells and signs of their plausible exhaustion in IgA nephropathy [20], a disease that shares certain pathophysiological features with IgAV [41]. In paediatric IgAV, NK cells might be implicated in nephritis development, as CD56^+^ NK cells expressing the activating receptor NKp44 were identified within glomeruli, while perforin-expressing CD56^+^ cells were observed in the tubular compartment [42]. In IgA nephropathy, NK cells have been shown to exert cytotoxic effects on glomerular endothelial cells, a mechanism thought to contribute to haematuria [43]. It is conceivable that NK cells in IgAV are initially activated and contribute to kidney injury, eventually leading to their exhaustion and reduced functional capacity in peripheral blood. Of interest, NK cells constitute a significant proportion (25%) of lymphocytes in the kidney [44] and are involved in the pathophysiology of several kidney diseases. Notably, a reduction in NK cell numbers and cytotoxic activity has also been reported in other vasculitides, such as Behçet’s disease [45], Takayasu’s arteritis [46], and granulomatosis with polyangiitis [47]. Furthermore, the impaired cytotoxic function of NK cells is not limited only to vasculitis, and was recently reported also in other autoimmune and rheumatic diseases, such as rheumatoid arthritis [48], systemic lupus erythematosus [49], systemic sclerosis [50], Sjögren’s disease [51] and type I diabetes mellitus [52]. Exhausted NK cells in autoimmune diseases may exhibit impaired clearance of stressed or damaged cells, potentially leading to the accumulation of cellular debris and the subsequent activation of immune responses against self-antigens.

The presence of interferons triggers the expression of interferon-induced genes constituting an interferon signature observed in patients with systemic connective tissue diseases, such as systemic lupus erythematosus, Sjögren’s disease, certain inflammatory myopathies, and systemic sclerosis [53]. Our study reports increased expression of multiple interferon-induced genes only in sl-IgAV patients. Recent studies [20, 54] have identified an increased expression of interferon signature genes such as IFI6, IFI44L, and IFITM3 in PBMCs of patients with IgA nephropathy. One of the primary functions of type I interferon, is to inhibit viral and bacterial infections [55, 56], which are considered potential triggers of IgAV [57, 58]. In our cohort, 3 out of 6 patients with sl-IgAV and 2 out of 5 patients with IgAVN reported recent or ongoing infections. Because infections are one of the triggers of IgAV, further analyses are needed to clarify whether the activation of the interferon signature found in our study is an integral part of IgAV or merely a reflection of an underlying (sub)clinical infection. Importantly, type I interferon directly and indirectly regulate NK cell maturation, homeostasis, and activation, and impaired type I interferon signalling has been associated with a substantial loss of mature NK cell function [59]. Even in chronic infections type I interferon regulates NK cytotoxicity [60] implying that the lack of interferon signature in IgAVN might partially explain the differences observed in NK cell function of sl-IgAV and IgAVN.

Importantly, leukocytes and PBMCs were collected from consecutively enrolled treatment-naïve IgAV patients, ensuring that the pathological processes observed were unaltered by immunomodulatory therapy. One limitation of our study is the absence of renal biopsy data, as biopsies were not clinically indicated in these patients. IgAVN diagnosis was established based on pathological urine analysis and subsequent improvement in renal function following immunomodulatory treatment. Another limitation is the small number of patients included in the RNA sequencing, however we confirmed reduced NK cells’ cytotoxic function in IgAVN patients with different in vitro assays on an additional larger cohort of adult IgAV patients. Incubation of NK cells with calcein-AM-labelled K562 cells showed a decreased cytotoxicity in IgAVN compared to sl-IgAV patients, however healthy NK cells presented median cytotoxic activity between IgVN and sl-IgAV with substantial variability between individuals. It remains an open question whether HC individuals with greater in vitro NK cell cytotoxicity may differ immunologically from those with lower NK activity when in pathological conditions. Compared to studies analysing NK cells in the whole blood [61, 62], we identified lower % of CD56^dim^CD16^brigh^ NK cells, which might be due to freezing of PBMCs.

Our analyses show changes in NK cell function in IgAVN patients, however confirmatory studies and biological significance of this needs to be further evaluated. We hypothesize that NK cells in IgAVN undergo initial activation, followed by exhaustion and decreased cytotoxic and immunomodulatory function. Relationship of NK cell exhaustion with the disease progression and long-term prognosis in IgAVN, warrants further investigation. Additionally, a protective role of NK in some autoimmune diseases was shown, as the adaptive transfer of NK cells delayed the onset of autoimmunity [63]. Targeting pathways involved in NK cell activation and function may therefore help restore NK cell function and lead to novel therapeutic approaches for IgAVN patients.

**Table 1:** Demographic and clinical characteristics of IgAV patients, IgAVN, sl-IgAV and HC included in RNA sequencing

Characteristics	IgAV (*n* = 6)	IgAVN (*n* = 3)	sl-IgAV (*n* = 3)	HC (*n* = 3)
Age*	66.5 (56.6–76.7)	66.6 (66.3–74.2)	64.0 (34.2–84.2)	53.0 (48.0–57.5)
Sex	1 M, 5 F	3 F	1 M, 2 F	3 F
BMI*	30.4 (24.6–35.9)	31.8 (26.0–40.0)	29.1 (20.4–34.5)	/
Symptom duration (day)*	5.5 (3.5–38)	5 (2–14)	6 (4–110)	/
Skin purpura	6	3	3	/
Daily proteinuria (g/day)*	0.351 (0.069–0.793)	0.474 (0.076–1.09)	0.228 (0.048–0.693)	/
Hematuria	3 microhematuria	3 microhematuria	0	/
CRP (g/l)*	51.5 (27.8–135.8)	58 (32–198)	45 (15–115)	/
SAA (µg/ml)	38.4 (0–175)	302 (92.5–933)	38.4 (0.00–598.0)	/
Number of lymphocytes (10^9^/l)*	1.83 (1.08–2.62)	1.58 (0.75–2.57)	2.08 (1.19–2.76)	/
Number of neutrophils (10^9^/l)*	5.12 (4.12–6.19)	4.5 (4.47–6.94)	7.46 (4.31–10.44)	/
BVAS*	7 (2–11)	8 (8–20)	2 (2–6)	/
Concurrent infection	1	0	1	/
Prior infection	2	1	1	/

IgAV, immunoglobulin A vasculitis; IgAVN, IgAV with renal involvement, sl-IgAV, skin-limited IgAV; HC, healthy controls; M, male; F, female; BMI, body mass index; CRP, C-reactive protein; SAA, serum amyloid A; BVAS, Birmingham vasculitis activity score; * median (IQR)

**Table 2:** Number of DEGs (|log2(FC)| ≥ 1 and p-adjusted value (p-adj) ≤ 0.05) in IgAV patients vs. healthy controls (HC), IgAVN vs. HC, sl-IgAV vs. HC and IgAVN vs. sl-IgAV

Comparison	Number DEGs	Up-regulated	Down-regulated
IgAV vs. HC	174	147	27
IgAVN vs. HC	234	181	53
sl-IgAV vs. HC	121	90	31
IgAVN vs. sl-IgAV	32	26	6

IgAV, immunoglobulin A vasculitis; IgAVN, IgAV withrenal involvement, sl-IgAV, skin-limited IgAV; HC, healthy controls; DEGs, differentially expressed genes

**Table 3:** Demographic and clinical characteristics of IgAV patients and HC included in NK cells analysis

Characteristics	IgAV (*n* = 16)	IgAVN (*n* = 10)	sl-IgAV (*n* = 6)	HC (*n* = 12)
Age*	68.7 (52.6–81.9)	71.0 (60.4–82.2)	60.7 (49.4–74.3)	59.7 (55.3–70.6)
Sex	7 F, 9 M	3 F, 7 M	4 F, 2 M	9 F, 3 M
BMI*	26.7 (24.1–32.1)	26.6 (23.7–30.3)	28.3 (24.6–38.3)	/
Symptom duration (day)*	10 (4–19.3)	8.5 (2.5–11)	20 (4.75–67.5)	/
Skin purpura	16	10	6	/
Daily proteinuria (g/day)*	0.210 (0.120–0.697)	0.274 (0.193–1.565)	0.095 (0.0375–0.193)	/
Hematuria	10 microhematuria, 1 macrohematuria	8 microhematuria, 1 macrohematuria	2 microhematuria	/
CRP (g/l)*	21.5 (5.25–37.5)	27.5 (7.75–43.8)	11.5 (4–40.75)	/
SAA (µg/ml)*	38.4 (0–175)	42.7 (15.4–317.5)	18.25 (0.00–92.1)	/
Number of lymphocytes (10^9^/l)*	1.71 (1.06–2.47)	1.87 (0.913–2.46)	1.58 (1.07–2.83)	/
Number of neutrophils (10^9^/l)*	5.12 (4.12–6.19)	5.22 (3.96–7.67)	5.03 (3.98–5.5)	/
BVAS*	7 (2–10)	9.5 (7.5–10.5)	2 (2–5.25)	/
Concurrent infection	3	2	1	/
Prior infection	7	5	2	/

IgAV, immunoglobulin A vasculitis; IgAVN, IgAV with renal involvement, sl-IgAV, skin-limited IgAV; HC, healthy controls; M, male; F, female; BMI, body mass index; CRP, C-reactive protein; SAA, serum amyloid A; BVAS, Birmingham vasculitis activity score; * median (IQR)

## Electronic supplementary material

Below is the link to the electronic supplementary material.

Supplementary Material 1

## Data Availability

No datasets were generated or analysed during the current study.

## Abbreviations

BMI: Body mass index
CCL: Chemokine (C-C motif) ligand
CRP: C-reactive protein
DEG: Differentially expressed gene
GIT: Gastrointestinal tract
HC: Healthy controls
IFN: Interferon
IgAV: Immunoglobulin A vasculitis
IgAVN: IgAV-with renal involvement
IL: Interleukin
KEGG: Kyoto Encyclopedia of Genes and Genomes
LU: Lytic units
MIP: Macrophage inflammatory protein
NK cells: Natural killer cells
ORA: Over-representation analysis
PBMC: Peripheral blood mononuclear cells
PBS: Phosphate-buffered saline
PCA: Principal component analysis
PHA: Phytohemagglutinin
qPCR: Quantitative polymerase chain reaction
RNA-Seq: RNA-sequencing
sl-IgAV: Skin-limited IgAV
TNF: Tumor necrosis factor
