# Supplementary material for: Exhausted natural killer cells in adult IgA vasculitis
Source: Arthritis Res Ther. 2025 Apr 23;27:95. doi: 10.1186/s13075-025-03559-y (PMC12016069; doi:10.1186/s13075-025-03559-y)
Supplement: Supplementary file 1 — Supplementary Material 1 [file 13075_2025_3559_MOESM1_ESM.docx]

Supplementary Material

Figure S1

**
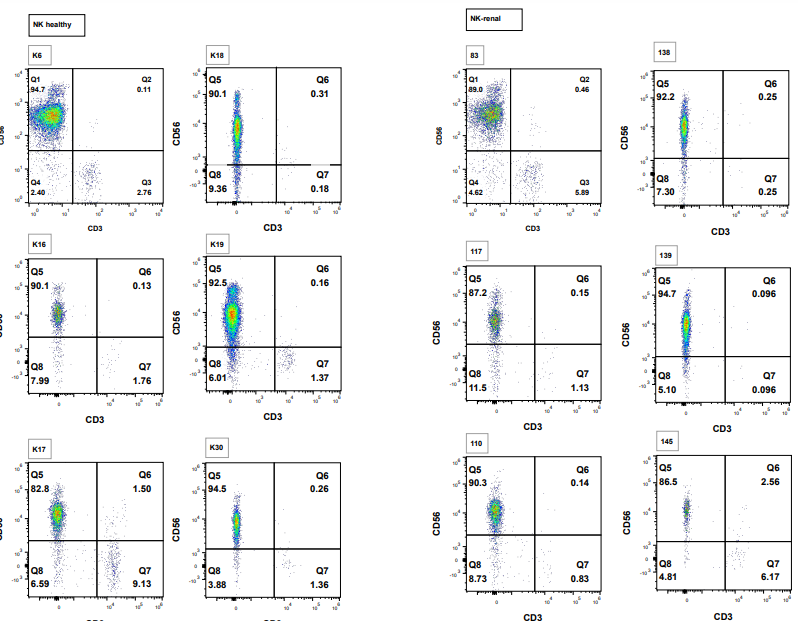
**

**
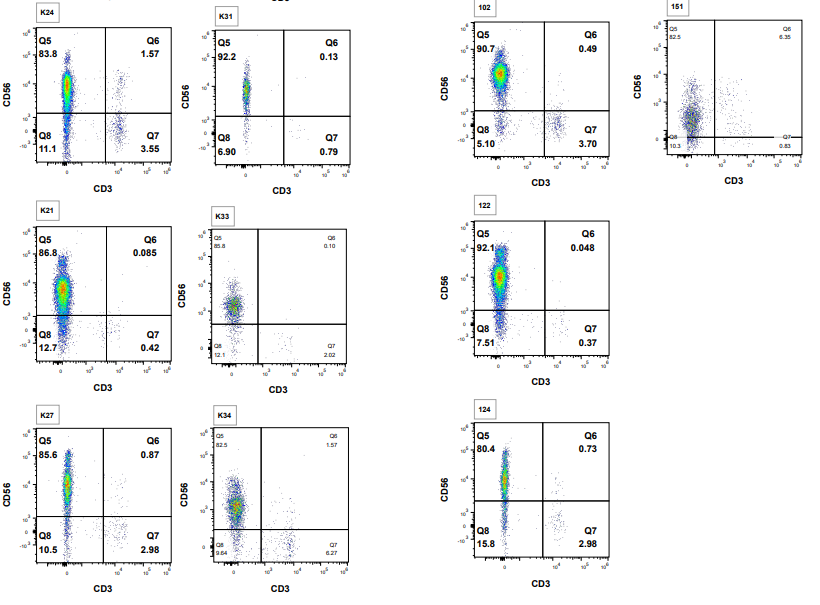
**

**
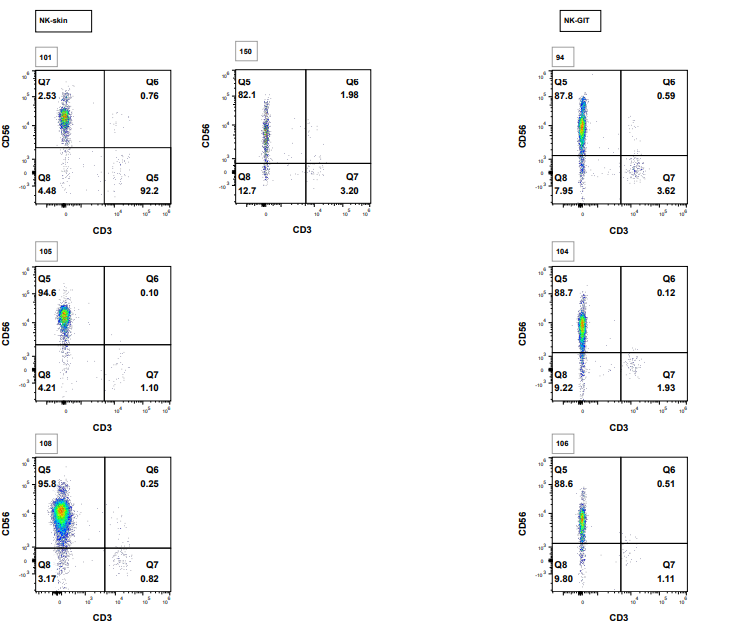
**

**
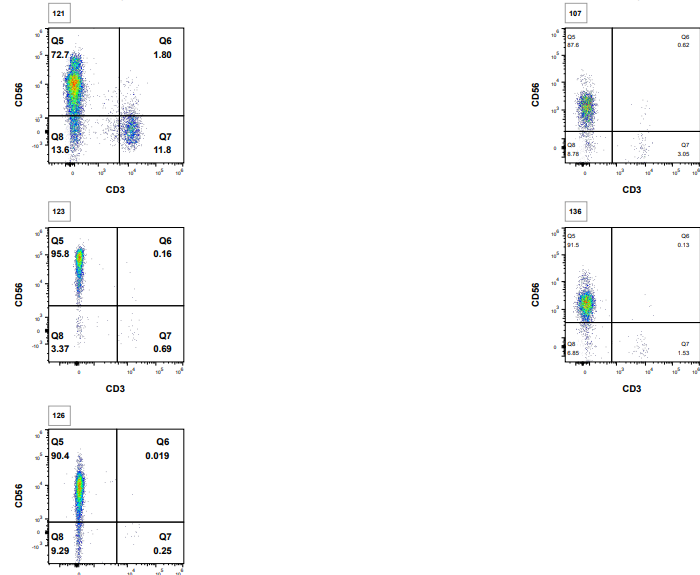
**

**Figure S1.** Determining purity of isolated NK-cells

NK cells, natural killer cells

**Figure S2**

**(a)** representative HC sample **(b)** representative HC sample after stimulation


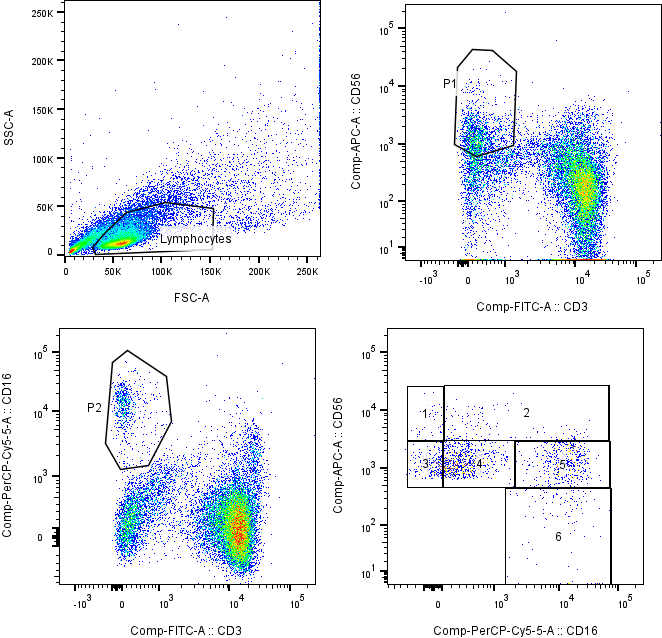

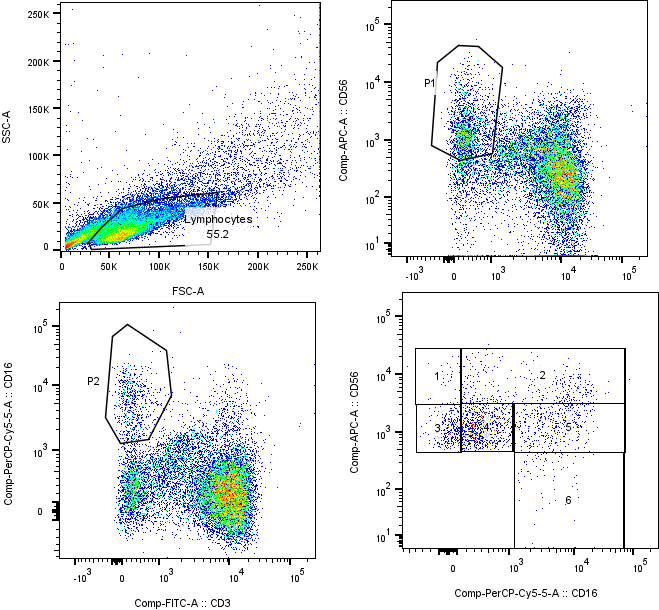


**(c)** representative IgAVN sample **(d)** representative IgAVN sample after stimulation


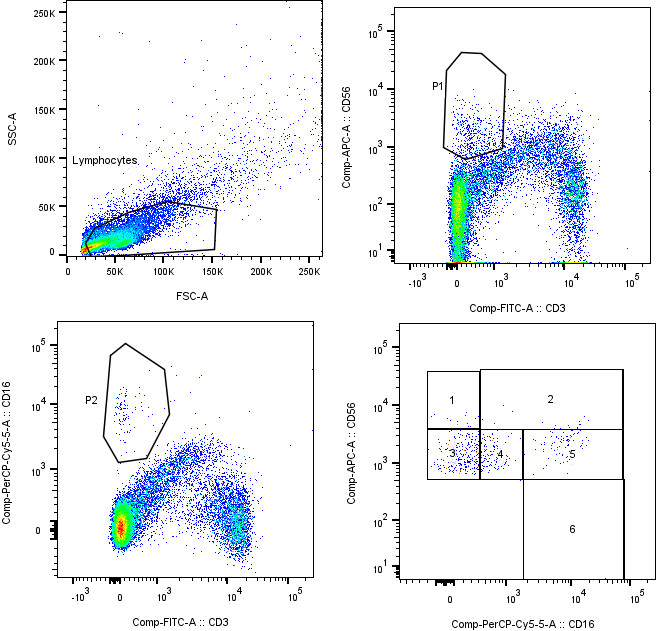

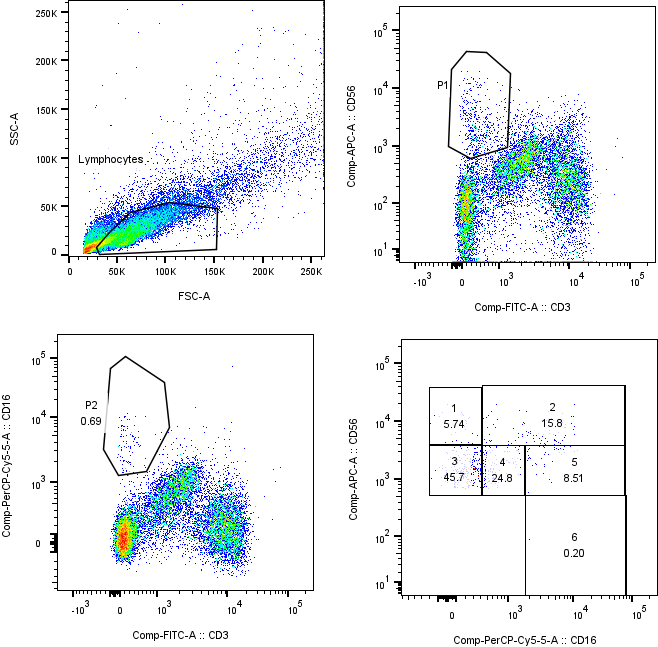


**Figure S2.** Gating strategy for the identification of NK cell subpopulations. Among lymphocytes identified by size and complexity (FSC vs SSC), NK cells were identified by the expression of CD56, CD16 and the absence of CD3 in a pseudocolour dot plot CD56 vs CD3 (p1) or CD16 vs CD3 (p2). Among all (p1+p2) NK cells 6 subpopulations were identified: CD56^bright^CD16^−^, CD56^bright^CD16^dim^, CD56^dim^CD16^−^, CD56^dim^CD16^dim^, CD56^dim^CD16^bright^, and CD56^−^CD16^bright^ as presented in gates 1, 2, 3, 4, 5, and 6, respectively. **(a)** A representative HC sample PBMC analysis is presented and **(b)** the same sample after stimulation. A representative IgAVN sample PBMC analysis is presented **(c)** and the same sample after stimulation **(d)**.

NK cells, natural killer cells; FSC, forward scatter; SSC, side scatter.

**Figure S3**

1. representative HC sample

**
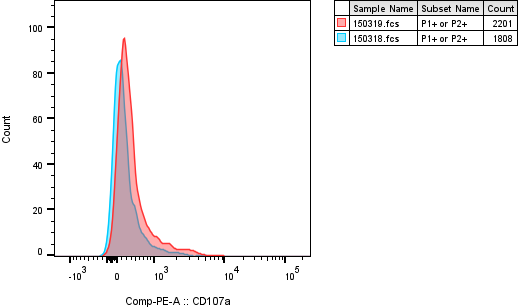

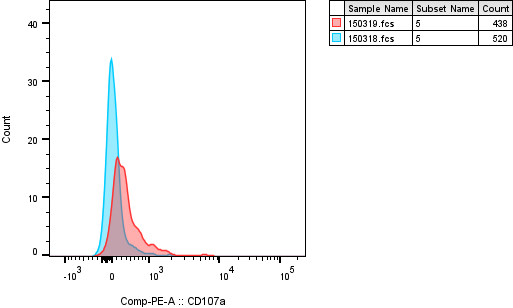
**

1. representative IgAVN sample


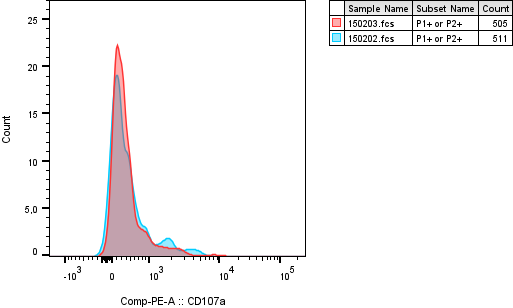

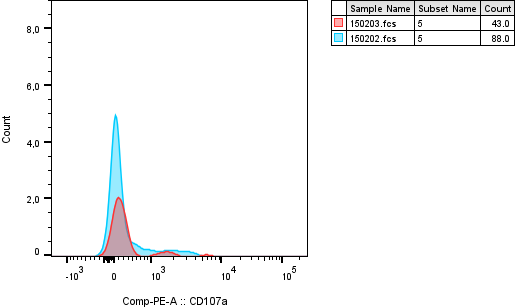


**Figure S3.** CD107a exposure on NK. **(a)** Representative overlay histograms for unstimulated (blue) and stimulated (red) HC - in all NK (p1+p2 population from FigS2) left and in cytotoxic, CD56dimCD16bright population (p5 from Fig S2) right. **(b)** Representative overlay histograms for unstimulated (blue) and stimulated (red) IgAV – in all NK (p1+p2 population from FigS2) left; in cytotoxic, CD56dimCD16bright population (p5 from Fig S2) right.

**Figure S4**


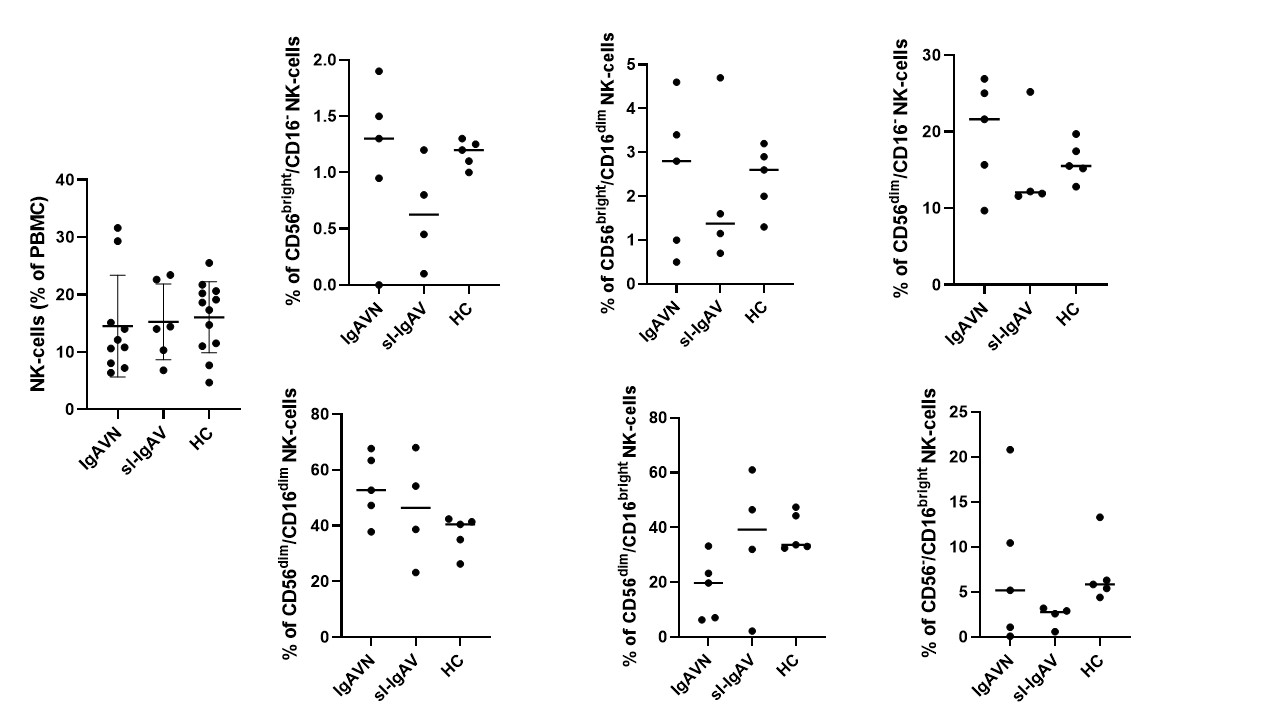


**Figure S4.** The % of NK cells and % of their 6 subtypes were not significantly changed between IgAVN, sl-IgAV and HC.

Data are expressed as medians of each group. NK cells, natural killer cells; PBMC, peripheral blood mononuclear cells; IgAV, immunoglobulin A vasculitis; HC, healthy controls; IgAVN, IgAV with renal involvement; sl-IgAV, skin-limited IgAV.

**Figure S5**


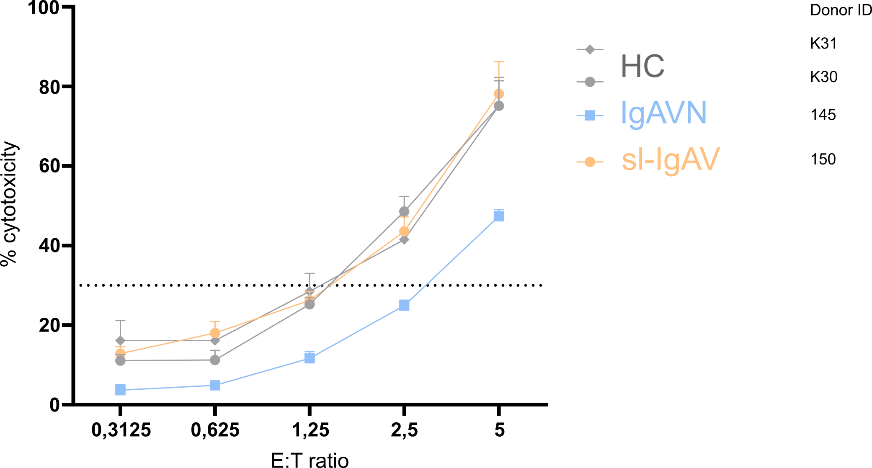


**Figure S5** Representative plot for % cytotoxicity versus E:T ratio.

E, effector cells; T, target cells.

**Figure S6**

1. **(b)**

**Figure S6 (a)** CD107a expression was increased on NK cells isolated from IgAVN patients before stimulation, although not reaching statistical significance. Data are expressed as medians of each group. NK cells, natural killer cells; IgAV, immunoglobulin A vasculitis; HC, healthy controls; IgAVN, IgAV with renal involvement; sl-IgAV, skin-limited IgAV, **(b)** Intracellular perforin was significantly decreased in NK cells from IgAVN patients compared to HC before stimulation as calculated with Kruskal-Wallis test followed by Dunn’s multiple comparison test.

**Figure S7**

**Figure S7** The expression of granzyme B between IgAV patients subgroups and HC was not changed before and after stimulation. Data are expressed as medians of each group. NK cells, natural killer cells; IgAV, immunoglobulin A vasculitis; HC, healthy controls; IgAVN, IgAV with renal involvement; sl-IgAV, skin-limited IgAV.

**Table S1.** Sequences of primers for qPCR

| Gene | Forward primer 5' to 3' | Reverse primer 3' to 5' |
| --- | --- | --- |
| GAPDH | CAGTGGCAAAGTGGAGATT | CTTGACTGTGCCGTTGAA |
| GBP1 | AGGAGTTCCTTCAAAGATGTGGA | GCAACTGGACCCTGTCGTT |
| GBP5 | ACATTAGTTCTGCTTGACACCG | GCTGCTCAGTAAGAGTGCCAG |
| IFIT3 | CAGCCATCATGAGTGAGGTC | TAAGTTCCAGGTGAAATGGCA |

**Table S2:** Differentially expressed genes in IgAVN patients compared to HC

|  | baseMean | log2FoldChange | lfcSE | pvalue | padj | symbol | ENTREZID | ENSEMBL |
| --- | --- | --- | --- | --- | --- | --- | --- | --- |
| ENSG00000235657 | 3409,43 | 15,44389 | 4,886879 | 1,22E-06 | 0,000254 | HLA-A | 3105 | ENSG00000235657 |
| ENSG00000226260 | 1061,362 | 14,83044 | 3,25816 | 3,28E-29 | 2,42E-25 | HLA-DRA | 3122 | ENSG00000226260 |
| ENSG00000204592 | 12721,23 | 12,81587 | 0,51971 | 1,07E-132 | 1,58E-128 | HLA-E | 3133 | ENSG00000204592 |
| ENSG00000225201 | 60,65663 | 9,99039 | 3,074027 | 3,12E-09 | 1,84E-06 | HLA-E | 3133 | ENSG00000225201 |
| ENSG00000241386 | 40,9313 | 9,500968 | 2,850414 | 2,88E-11 | 3,27E-08 | HLA-DOB | 3112 | ENSG00000241386 |
| ENSG00000236884 | 960,2541 | 8,78352 | 1,963732 | 1,93E-07 | 5,91E-05 | HLA-DRB1 | 3123 | ENSG00000236884 |
| ENSG00000235844 | 16,49596 | 7,799303 | 2,784257 | 3,49E-07 | 9,71E-05 | HLA-DPA1 | 3113 | ENSG00000235844 |
| ENSG00000283802 | 71,6126 | 7,080234 | 1,559257 | 5,65E-07 | 0,000141 | ADAMTS2 | 9509 | ENSG00000283802 |
| ENSG00000167613 | 16,8216 | 5,728876 | 1,948706 | 0,000104 | 0,008582 | LAIR1 | 3903 | ENSG00000167613 |
| ENSG00000277585 | 19,34311 | 5,117371 | 1,421657 | 3,42E-05 | 0,003632 | MUC4 | 4585 | ENSG00000277585 |
| ENSG00000174844 | 9,623842 | 3,787101 | 1,376178 | 0,000219 | 0,014817 | DNAH12 | 201625 | ENSG00000174844 |
| ENSG00000189221 | 19,69355 | 3,776707 | 1,15158 | 5,03E-05 | 0,004819 | MAOA | 4128 | ENSG00000189221 |
| ENSG00000169877 | 62,84294 | 3,402012 | 0,680601 | 1,82E-08 | 8,41E-06 | AHSP | 51327 | ENSG00000169877 |
| ENSG00000196611 | 26,23109 | 3,355584 | 1,445521 | 0,000521 | 0,029074 | MMP1 | 4312 | ENSG00000196611 |
| ENSG00000187010 | 144,9236 | 3,230829 | 0,529833 | 4,90E-11 | 4,82E-08 | RHD | 6007 | ENSG00000187010 |
| ENSG00000103184 | 66,92798 | 3,035516 | 0,464896 | 3,39E-12 | 5,00E-09 | SEC14L5 | 9717 | ENSG00000103184 |
| ENSG00000005961 | 8272,919 | 3,011884 | 0,487951 | 3,50E-11 | 3,68E-08 | ITGA2B | 3674 | ENSG00000005961 |
| ENSG00000100583 | 19,29484 | 3,008738 | 1,477342 | 0,00086 | 0,040232 | SAMD15 | 161394 | ENSG00000100583 |
| ENSG00000004939 | 1272,535 | 3,006354 | 0,479432 | 1,61E-11 | 2,15E-08 | SLC4A1 | 6521 | ENSG00000004939 |
| ENSG00000158578 | 1196,609 | 2,967821 | 0,655352 | 2,32E-07 | 6,83E-05 | ALAS2 | 212 | ENSG00000158578 |
| ENSG00000257017 | 901,9133 | 2,900694 | 0,510598 | 5,83E-10 | 3,91E-07 | HP | 3240 | ENSG00000257017 |
| ENSG00000204936 | 266,7875 | 2,886012 | 0,683728 | 9,38E-07 | 0,000203 | CD177 | 57126 | ENSG00000204936 |
| ENSG00000101335 | 541,8842 | 2,83058 | 0,365049 | 4,41E-16 | 2,16E-12 | MYL9 | 10398 | ENSG00000101335 |
| ENSG00000110693 | 124,1302 | 2,787644 | 0,628901 | 3,65E-07 | 9,80E-05 | SOX6 | 55553 | ENSG00000110693 |
| ENSG00000223609 | 242,3514 | 2,764412 | 0,791101 | 1,62E-05 | 0,002044 | HBD | 3045 | ENSG00000223609 |
| ENSG00000259207 | 4534,922 | 2,591192 | 0,472143 | 1,87E-09 | 1,15E-06 | ITGB3 | 3690 | ENSG00000259207 |
| ENSG00000138722 | 493,2483 | 2,583191 | 0,370941 | 1,58E-13 | 3,88E-10 | MMRN1 | 22915 | ENSG00000138722 |
| ENSG00000003436 | 68,0848 | 2,555235 | 0,661716 | 3,98E-06 | 0,000674 | TFPI | 7035 | ENSG00000003436 |
| ENSG00000109272 | 364,964 | 2,497003 | 0,829738 | 8,21E-05 | 0,007075 | PF4V1 | 5197 | ENSG00000109272 |
| ENSG00000069535 | 28,09144 | 2,466129 | 0,778572 | 5,88E-05 | 0,005555 | MAOB | 4129 | ENSG00000069535 |
| ENSG00000173210 | 508,6107 | 2,385455 | 0,384042 | 2,34E-11 | 2,88E-08 | ABLIM3 | 22885 | ENSG00000173210 |
| ENSG00000134668 | 86,30389 | 2,383884 | 0,704167 | 2,62E-05 | 0,003085 | SPOCD1 | 90853 | ENSG00000134668 |
| ENSG00000140682 | 53,25211 | 2,362084 | 0,702479 | 2,94E-05 | 0,00331 | TGFB1I1 | 7041 | ENSG00000140682 |
| ENSG00000136842 | 39,897 | 2,312992 | 0,902547 | 0,000333 | 0,020814 | TMOD1 | 7111 | ENSG00000136842 |
| ENSG00000169247 | 242,0033 | 2,290792 | 0,609986 | 6,42E-06 | 0,001005 | SH3TC2 | 79628 | ENSG00000169247 |
| ENSG00000100985 | 5756,65 | 2,288562 | 0,311981 | 1,04E-14 | 3,08E-11 | MMP9 | 4318 | ENSG00000100985 |
| ENSG00000167100 | 43,36223 | 2,284618 | 0,779915 | 9,85E-05 | 0,008252 | SAMD14 | 201191 | ENSG00000167100 |
| ENSG00000251158 | 466,9981 | 2,181256 | 0,815492 | 0,000225 | 0,0151 | LOC728506 | 728506 | ENSG00000251158 |
| ENSG00000110203 | 210,5779 | 2,178463 | 0,764915 | 0,000136 | 0,010463 | FOLR3 | 2352 | ENSG00000110203 |
| ENSG00000087237 | 68,11972 | 2,176054 | 0,580953 | 6,99E-06 | 0,001051 | CETP | 1071 | ENSG00000087237 |
| ENSG00000145685 | 70,13842 | 2,173715 | 0,462271 | 1,07E-07 | 3,74E-05 | LHFPL2 | 10184 | ENSG00000145685 |
| ENSG00000154529 | 96,64805 | 2,147295 | 0,691215 | 6,48E-05 | 0,0059 | CNTNAP3B | 728577 | ENSG00000154529 |
| ENSG00000143416 | 65,27501 | 2,133558 | 0,618922 | 2,07E-05 | 0,002565 | SELENBP1 | 8991 | ENSG00000143416 |
| ENSG00000108950 | 105,0887 | 2,132924 | 1,062994 | 0,000956 | 0,043233 | FAM20A | 54757 | ENSG00000108950 |
| ENSG00000012779 | 2202,387 | 2,111317 | 0,587832 | 1,27E-05 | 0,001723 | ALOX5 | 240 | ENSG00000012779 |
| ENSG00000166086 | 669,216 | 2,110341 | 0,504556 | 1,18E-06 | 0,000248 | JAM3 | 83700 | ENSG00000166086 |
| ENSG00000140479 | 293,9444 | 2,10511 | 0,590919 | 1,39E-05 | 0,001858 | PCSK6 | 5046 | ENSG00000140479 |
| ENSG00000166091 | 108,0877 | 2,097493 | 0,475603 | 4,43E-07 | 0,000117 | CMTM5 | 116173 | ENSG00000166091 |
| ENSG00000284874 | 180,5696 | 2,097285 | 0,445128 | 1,09E-07 | 3,75E-05 | SEPT5-GP1BB | 1,01E+08 | ENSG00000284874 |
| ENSG00000166947 | 109,2267 | 2,088207 | 0,650589 | 4,42E-05 | 0,004406 | EPB42 | 2038 | ENSG00000166947 |
| ENSG00000118514 | 15,39378 | 2,081509 | 0,975068 | 0,000862 | 0,040237 | ALDH8A1 | 64577 | ENSG00000118514 |
| ENSG00000204424 | 134,6231 | 2,078324 | 0,553194 | 6,69E-06 | 0,00102 | LY6G6F | 259215 | ENSG00000204424 |
| ENSG00000175928 | 61,71566 | 2,063833 | 0,579203 | 1,48E-05 | 0,001954 | LRRN1 | 57633 | ENSG00000175928 |
| ENSG00000187800 | 194,3395 | 2,045994 | 0,475536 | 7,11E-07 | 0,000166 | PEAR1 | 375033 | ENSG00000187800 |
| ENSG00000263155 | 70,03763 | 2,044095 | 0,65795 | 6,63E-05 | 0,005998 | MYZAP | 1,01E+08 | ENSG00000263155 |
| ENSG00000166963 | 556,718 | 2,033089 | 0,554708 | 9,83E-06 | 0,001435 | MAP1A | 4130 | ENSG00000166963 |
| ENSG00000164116 | 195,3209 | 2,014107 | 0,335383 | 9,17E-11 | 7,65E-08 | GUCY1A1 | 2982 | ENSG00000164116 |
| ENSG00000161911 | 492,1379 | 2,013572 | 0,38985 | 1,10E-08 | 5,99E-06 | TREML1 | 340205 | ENSG00000161911 |
| ENSG00000143595 | 89,28415 | 2,012216 | 0,598013 | 2,79E-05 | 0,003217 | AQP10 | 89872 | ENSG00000143595 |
| ENSG00000174175 | 492,7174 | 1,999622 | 0,330796 | 7,28E-11 | 6,71E-08 | SELP | 6403 | ENSG00000174175 |
| ENSG00000169704 | 333,5801 | 1,991678 | 0,400217 | 2,93E-08 | 1,31E-05 | GP9 | 2815 | ENSG00000169704 |
| ENSG00000203618 | 529,7848 | 1,965084 | 0,452861 | 6,17E-07 | 0,000147 | GP1BB | 2812 | ENSG00000203618 |
| ENSG00000118520 | 437,2955 | 1,964774 | 0,588292 | 3,13E-05 | 0,003416 | ARG1 | 383 | ENSG00000118520 |
| ENSG00000117400 | 217,2149 | 1,959153 | 0,452162 | 6,18E-07 | 0,000147 | MPL | 4352 | ENSG00000117400 |
| ENSG00000006638 | 140,1641 | 1,94427 | 0,407612 | 8,30E-08 | 3,06E-05 | TBXA2R | 6915 | ENSG00000006638 |
| ENSG00000162722 | 696,1557 | 1,937806 | 0,349303 | 1,37E-09 | 8,77E-07 | TRIM58 | 25893 | ENSG00000162722 |
| ENSG00000119242 | 78,7396 | 1,93129 | 0,567615 | 2,56E-05 | 0,003044 | CCDC92 | 80212 | ENSG00000119242 |
| ENSG00000163736 | 9440,55 | 1,926613 | 0,375356 | 1,79E-08 | 8,41E-06 | PPBP | 5473 | ENSG00000163736 |
| ENSG00000112299 | 309,4889 | 1,900204 | 0,582534 | 4,05E-05 | 0,004092 | VNN1 | 8876 | ENSG00000112299 |
| ENSG00000163898 | 47,7003 | 1,897079 | 0,56735 | 3,04E-05 | 0,003346 | LIPH | 200879 | ENSG00000163898 |
| ENSG00000185245 | 1070,982 | 1,88224 | 0,370673 | 1,77E-08 | 8,41E-06 | GP1BA | 2811 | ENSG00000185245 |
| ENSG00000095303 | 1243,374 | 1,872886 | 0,387107 | 6,11E-08 | 2,44E-05 | PTGS1 | 5742 | ENSG00000095303 |
| ENSG00000137198 | 320,1253 | 1,858684 | 0,432269 | 7,29E-07 | 0,000168 | GMPR | 2766 | ENSG00000137198 |
| ENSG00000124491 | 8851,23 | 1,844943 | 0,274043 | 8,53E-13 | 1,49E-09 | F13A1 | 2162 | ENSG00000124491 |
| ENSG00000154146 | 566,3342 | 1,837103 | 0,451828 | 2,04E-06 | 0,000385 | NRGN | 4900 | ENSG00000154146 |
| ENSG00000163737 | 2194,034 | 1,80987 | 0,303718 | 1,25E-10 | 9,22E-08 | PF4 | 5196 | ENSG00000163737 |
| ENSG00000278555 | 171,7272 | 1,80978 | 0,63796 | 0,000153 | 0,011231 | LILRB4 | 11006 | ENSG00000278555 |
| ENSG00000250138 | 8939,167 | 1,797332 | 0,607894 | 0,000119 | 0,009557 | LOC728488 | 728488 | ENSG00000250138 |
| ENSG00000184500 | 208,3431 | 1,796552 | 0,538816 | 3,31E-05 | 0,003558 | PROS1 | 5627 | ENSG00000184500 |
| ENSG00000184792 | 292,1451 | 1,790911 | 0,686463 | 0,000284 | 0,018366 | OSBP2 | 23762 | ENSG00000184792 |
| ENSG00000049323 | 1309,448 | 1,78605 | 0,377766 | 9,86E-08 | 3,55E-05 | LTBP1 | 4052 | ENSG00000049323 |
| ENSG00000120885 | 3792,684 | 1,784405 | 0,369653 | 6,94E-08 | 2,62E-05 | CLU | 1191 | ENSG00000120885 |
| ENSG00000113140 | 2132,784 | 1,744888 | 0,407839 | 8,56E-07 | 0,000188 | SPARC | 6678 | ENSG00000113140 |
| ENSG00000122786 | 143,7572 | 1,732838 | 0,589171 | 0,00011 | 0,009025 | CALD1 | 800 | ENSG00000122786 |
| ENSG00000149564 | 216,5333 | 1,732157 | 0,507741 | 2,52E-05 | 0,003019 | ESAM | 90952 | ENSG00000149564 |
| ENSG00000278563 | 787,3209 | 1,720499 | 0,445677 | 4,80E-06 | 0,000778 | MGAM2 | 93432 | ENSG00000278563 |
| ENSG00000205038 | 540,4604 | 1,717669 | 0,604248 | 0,00015 | 0,011128 | PKHD1L1 | 93035 | ENSG00000205038 |
| ENSG00000163221 | 1398,817 | 1,694497 | 0,371982 | 2,23E-07 | 6,72E-05 | S100A12 | 6283 | ENSG00000163221 |
| ENSG00000102362 | 120,443 | 1,679013 | 0,421167 | 2,93E-06 | 0,000527 | SYTL4 | 94121 | ENSG00000102362 |
| ENSG00000029534 | 1243,697 | 1,676132 | 0,382573 | 5,40E-07 | 0,000137 | ANK1 | 286 | ENSG00000029534 |
| ENSG00000145335 | 2126,398 | 1,67508 | 0,242664 | 2,66E-13 | 5,60E-10 | SNCA | 6622 | ENSG00000145335 |
| ENSG00000163430 | 132,9334 | 1,660589 | 0,788853 | 0,000959 | 0,043233 | FSTL1 | 11167 | ENSG00000163430 |
| ENSG00000072422 | 173,3403 | 1,65305 | 0,394956 | 1,25E-06 | 0,000257 | RHOBTB1 | 9886 | ENSG00000072422 |
| ENSG00000132970 | 56,16144 | 1,635714 | 0,569482 | 0,000138 | 0,010544 | WASF3 | 10810 | ENSG00000132970 |
| ENSG00000158856 | 1034,536 | 1,631567 | 0,346128 | 1,13E-07 | 3,80E-05 | DMTN | 2039 | ENSG00000158856 |
| ENSG00000163359 | 293,7187 | 1,629105 | 0,393947 | 1,58E-06 | 0,00031 | COL6A3 | 1293 | ENSG00000163359 |
| ENSG00000158352 | 115,786 | 1,62301 | 0,69184 | 0,000574 | 0,030657 | SHROOM4 | 57477 | ENSG00000158352 |
| ENSG00000070182 | 627,4464 | 1,621762 | 0,567496 | 0,000152 | 0,01121 | SPTB | 6710 | ENSG00000070182 |
| ENSG00000108846 | 850,0555 | 1,603589 | 0,37322 | 7,89E-07 | 0,000179 | ABCC3 | 8714 | ENSG00000108846 |
| ENSG00000130821 | 189,3468 | 1,5814 | 0,646876 | 0,000449 | 0,025883 | SLC6A8 | 6535 | ENSG00000130821 |
| ENSG00000135862 | 113,2888 | 1,568396 | 0,677964 | 0,000606 | 0,031935 | LAMC1 | 3915 | ENSG00000135862 |
| ENSG00000008438 | 320,1962 | 1,560382 | 0,409636 | 6,06E-06 | 0,000961 | PGLYRP1 | 8993 | ENSG00000008438 |
| ENSG00000144893 | 118,5012 | 1,554492 | 0,45844 | 2,68E-05 | 0,003135 | MED12L | 116931 | ENSG00000144893 |
| ENSG00000125257 | 615,0558 | 1,550957 | 0,549363 | 0,00017 | 0,012084 | ABCC4 | 10257 | ENSG00000125257 |
| ENSG00000152952 | 97,36274 | 1,549042 | 0,693422 | 0,000741 | 0,036721 | PLOD2 | 5352 | ENSG00000152952 |
| ENSG00000125538 | 1214,189 | 1,54734 | 0,444611 | 2,15E-05 | 0,002639 | IL1B | 3553 | ENSG00000125538 |
| ENSG00000165626 | 59,94025 | 1,545823 | 0,726094 | 0,000928 | 0,042242 | BEND7 | 222389 | ENSG00000165626 |
| ENSG00000282607 | 8521,469 | 1,5412 | 0,314732 | 4,91E-08 | 2,07E-05 | MGAM | 8972 | ENSG00000282607 |
| ENSG00000022267 | 594,2316 | 1,537307 | 0,332546 | 1,79E-07 | 5,75E-05 | FHL1 | 2273 | ENSG00000022267 |
| ENSG00000112053 | 208,1026 | 1,533695 | 0,68246 | 0,000733 | 0,03651 | SLC26A8 | 116369 | ENSG00000112053 |
| ENSG00000108839 | 267,333 | 1,530672 | 0,430782 | 1,57E-05 | 0,002007 | ALOX12 | 239 | ENSG00000108839 |
| ENSG00000047648 | 209,2833 | 1,528178 | 0,453172 | 2,96E-05 | 0,00331 | ARHGAP6 | 395 | ENSG00000047648 |
| ENSG00000198478 | 259,245 | 1,526365 | 0,470471 | 4,72E-05 | 0,004635 | SH3BGRL2 | 83699 | ENSG00000198478 |
| ENSG00000288401 | 1453,104 | 1,506803 | 0,261706 | 4,38E-10 | 3,07E-07 | CTTN | 2017 | ENSG00000288401 |
| ENSG00000180549 | 914,87 | 1,503793 | 0,281318 | 4,62E-09 | 2,62E-06 | FUT7 | 2529 | ENSG00000180549 |
| ENSG00000082781 | 382,6046 | 1,501639 | 0,500714 | 0,000104 | 0,008582 | ITGB5 | 3693 | ENSG00000082781 |
| ENSG00000257335 | 20127,1 | 1,492867 | 0,311733 | 6,39E-08 | 2,48E-05 | MGAM | 8972 | ENSG00000257335 |
| ENSG00000146122 | 108,6682 | 1,492288 | 0,635781 | 0,000587 | 0,031158 | DAAM2 | 23500 | ENSG00000146122 |
| ENSG00000047597 | 167,4183 | 1,488339 | 0,508651 | 0,000129 | 0,010041 | XK | 7504 | ENSG00000047597 |
| ENSG00000186205 | 473,6309 | 1,488314 | 0,716678 | 0,001059 | 0,046059 | MTARC1 | 64757 | ENSG00000186205 |
| ENSG00000169313 | 151,8654 | 1,469978 | 0,328534 | 3,66E-07 | 9,80E-05 | P2RY12 | 64805 | ENSG00000169313 |
| ENSG00000081377 | 271,7193 | 1,452906 | 0,298902 | 5,67E-08 | 2,32E-05 | CDC14B | 8555 | ENSG00000081377 |
| ENSG00000065534 | 717,437 | 1,442796 | 0,291322 | 3,59E-08 | 1,56E-05 | MYLK | 4638 | ENSG00000065534 |
| ENSG00000184702 | 589,7803 | 1,437079 | 0,346534 | 1,57E-06 | 0,00031 | SEPTIN5 | 5413 | ENSG00000184702 |
| ENSG00000101162 | 12923,8 | 1,435391 | 0,316573 | 2,81E-07 | 7,98E-05 | TUBB1 | 81027 | ENSG00000101162 |
| ENSG00000277816 | 337,9715 | 1,40971 | 0,590585 | 0,000566 | 0,030441 | LILRB3 | 11025 | ENSG00000277816 |
| ENSG00000284096 | 1670,553 | 1,393937 | 0,38339 | 1,27E-05 | 0,001723 | PRKAR2B | 5577 | ENSG00000284096 |
| ENSG00000106714 | 3562,826 | 1,387122 | 0,447996 | 7,93E-05 | 0,006921 | CNTNAP3 | 79937 | ENSG00000106714 |
| ENSG00000133742 | 287,9344 | 1,383955 | 0,590184 | 0,000625 | 0,032552 | CA1 | 759 | ENSG00000133742 |
| ENSG00000164181 | 256,2953 | 1,376291 | 0,467671 | 0,000124 | 0,009816 | ELOVL7 | 79993 | ENSG00000164181 |
| ENSG00000185052 | 131,8611 | 1,365064 | 0,400352 | 2,86E-05 | 0,003238 | SLC24A3 | 57419 | ENSG00000185052 |
| ENSG00000205639 | 52,3865 | 1,338628 | 0,647041 | 0,001192 | 0,048834 | MFSD2B | 388931 | ENSG00000205639 |
| ENSG00000138798 | 547,426 | 1,338414 | 0,503098 | 0,000283 | 0,018366 | EGF | 1950 | ENSG00000138798 |
| ENSG00000110799 | 548,6857 | 1,337441 | 0,490015 | 0,000243 | 0,015994 | VWF | 7450 | ENSG00000110799 |
| ENSG00000013016 | 309,3961 | 1,323194 | 0,407139 | 4,89E-05 | 0,004713 | EHD3 | 30845 | ENSG00000013016 |
| ENSG00000144677 | 166,3093 | 1,304731 | 0,629225 | 0,001142 | 0,047557 | CTDSPL | 10217 | ENSG00000144677 |
| ENSG00000182732 | 79,33443 | 1,300746 | 0,469267 | 0,000217 | 0,014817 | RGS6 | 9628 | ENSG00000182732 |
| ENSG00000168497 | 1493,244 | 1,294046 | 0,293061 | 5,04E-07 | 0,00013 | CAVIN2 | 8436 | ENSG00000168497 |
| ENSG00000106070 | 342,5778 | 1,278469 | 0,583497 | 0,0009 | 0,041341 | GRB10 | 2887 | ENSG00000106070 |
| ENSG00000172572 | 112,0505 | 1,277221 | 0,413936 | 8,53E-05 | 0,007316 | PDE3A | 5139 | ENSG00000172572 |
| ENSG00000061918 | 366,0739 | 1,272185 | 0,37928 | 3,48E-05 | 0,003635 | GUCY1B1 | 2983 | ENSG00000061918 |
| ENSG00000140416 | 912,5861 | 1,253276 | 0,445152 | 0,000195 | 0,013493 | TPM1 | 7168 | ENSG00000140416 |
| ENSG00000119862 | 280,8692 | 1,23042 | 0,426094 | 0,000158 | 0,011481 | LGALSL | 29094 | ENSG00000119862 |
| ENSG00000215883 | 52,27767 | 1,215501 | 0,522542 | 0,000697 | 0,035334 | CYB5RL | 606495 | ENSG00000215883 |
| ENSG00000172159 | 169,2529 | 1,215358 | 0,568638 | 0,001055 | 0,046004 | FRMD3 | 257019 | ENSG00000172159 |
| ENSG00000283378 | 2007,059 | 1,21385 | 0,468625 | 0,000366 | 0,022495 | CNTNAP3C | 1E+08 | ENSG00000283378 |
| ENSG00000061676 | 294,6977 | 1,210278 | 0,335996 | 1,48E-05 | 0,001954 | NCKAP1 | 10787 | ENSG00000061676 |
| ENSG00000128245 | 895,4877 | 1,201443 | 0,421387 | 0,000167 | 0,012 | YWHAH | 7533 | ENSG00000128245 |
| ENSG00000143409 | 923,0124 | 1,199895 | 0,324197 | 1,03E-05 | 0,001494 | MINDY1 | 55793 | ENSG00000143409 |
| ENSG00000169902 | 181,784 | 1,17772 | 0,496192 | 0,000624 | 0,032552 | TPST1 | 8460 | ENSG00000169902 |
| ENSG00000101856 | 462,5631 | 1,171343 | 0,389492 | 0,000113 | 0,009182 | PGRMC1 | 10857 | ENSG00000101856 |
| ENSG00000135636 | 23602,62 | 1,170999 | 0,339259 | 2,81E-05 | 0,003217 | DYSF | 8291 | ENSG00000135636 |
| ENSG00000138772 | 1298,751 | 1,161316 | 0,498679 | 0,000707 | 0,035583 | ANXA3 | 306 | ENSG00000138772 |
| ENSG00000198019 | 1373,734 | 1,153089 | 0,444638 | 0,000384 | 0,023056 | FCGR1B | 2210 | ENSG00000198019 |
| ENSG00000074416 | 385,8938 | 1,136966 | 0,307324 | 1,08E-05 | 0,001531 | MGLL | 11343 | ENSG00000074416 |
| ENSG00000158457 | 375,287 | 1,128451 | 0,307193 | 1,17E-05 | 0,001625 | TSPAN33 | 340348 | ENSG00000158457 |
| ENSG00000280908 | 2012,755 | 1,112407 | 0,390248 | 0,00019 | 0,013263 | PADI4 | 23569 | ENSG00000280908 |
| ENSG00000103196 | 1194,113 | 1,112054 | 0,493202 | 0,00086 | 0,040232 | CRISPLD2 | 83716 | ENSG00000103196 |
| ENSG00000082146 | 389,1546 | 1,11096 | 0,322821 | 2,79E-05 | 0,003217 | STRADB | 55437 | ENSG00000082146 |
| ENSG00000035403 | 9389,416 | 1,110717 | 0,257042 | 8,46E-07 | 0,000188 | VCL | 7414 | ENSG00000035403 |
| ENSG00000120696 | 408,0882 | 1,105336 | 0,273905 | 2,82E-06 | 0,000514 | KBTBD7 | 84078 | ENSG00000120696 |
| ENSG00000008516 | 2920,601 | 1,096635 | 0,439891 | 0,000496 | 0,027908 | MMP25 | 64386 | ENSG00000008516 |
| ENSG00000162551 | 5672,051 | 1,074493 | 0,511041 | 0,001224 | 0,049994 | ALPL | 249 | ENSG00000162551 |
| ENSG00000066926 | 677,0695 | 1,071332 | 0,27341 | 4,61E-06 | 0,000756 | FECH | 2235 | ENSG00000066926 |
| ENSG00000170909 | 329,0239 | 1,071061 | 0,342323 | 8,09E-05 | 0,007018 | OSCAR | 126014 | ENSG00000170909 |
| ENSG00000187699 | 205,5683 | 1,063958 | 0,487168 | 0,001027 | 0,045356 | C2orf88 | 84281 | ENSG00000187699 |
| ENSG00000148926 | 414,9946 | 1,063397 | 0,360205 | 0,000142 | 0,010719 | ADM | 133 | ENSG00000148926 |
| ENSG00000059804 | 13651,72 | 1,060753 | 0,268942 | 4,38E-06 | 0,000734 | SLC2A3 | 6515 | ENSG00000059804 |
| ENSG00000107438 | 728,5468 | 1,058298 | 0,445165 | 0,000664 | 0,034242 | PDLIM1 | 9124 | ENSG00000107438 |
| ENSG00000148498 | 204,025 | 1,05577 | 0,484686 | 0,001048 | 0,045848 | PARD3 | 56288 | ENSG00000148498 |
| ENSG00000188191 | 132,0452 | 1,054371 | 0,462047 | 0,000828 | 0,039251 | PRKAR1B | 5575 | ENSG00000188191 |
| ENSG00000178726 | 974,641 | 1,042149 | 0,259806 | 3,25E-06 | 0,000564 | THBD | 7056 | ENSG00000178726 |
| ENSG00000121316 | 4275,657 | 1,04192 | 0,313005 | 4,09E-05 | 0,004101 | PLBD1 | 79887 | ENSG00000121316 |
| ENSG00000124588 | 413,2509 | 1,040201 | 0,393649 | 0,000347 | 0,021602 | NQO2 | 4835 | ENSG00000124588 |
| ENSG00000115828 | 1462,99 | 1,037279 | 0,264129 | 4,59E-06 | 0,000756 | QPCT | 25797 | ENSG00000115828 |
| ENSG00000163421 | 2096,939 | 1,036569 | 0,438188 | 0,000725 | 0,036364 | PROK2 | 60675 | ENSG00000163421 |
| ENSG00000138735 | 559,4145 | 1,033361 | 0,423902 | 0,000575 | 0,030657 | PDE5A | 8654 | ENSG00000138735 |
| ENSG00000281818 | 462,5153 | 1,02425 | 0,43934 | 0,000765 | 0,037453 | MANSC1 | 54682 | ENSG00000281818 |
| ENSG00000100504 | 13708,05 | 1,015922 | 0,344189 | 0,000149 | 0,011127 | PYGL | 5836 | ENSG00000100504 |
| ENSG00000064601 | 8698,696 | 1,014723 | 0,297012 | 3,03E-05 | 0,003346 | CTSA | 5476 | ENSG00000064601 |
| ENSG00000111644 | 186,312 | 1,010167 | 0,457369 | 0,001005 | 0,04477 | ACRBP | 84519 | ENSG00000111644 |
| ENSG00000225614 | 219,2338 | -1,00884 | 0,279228 | 1,56E-05 | 0,002007 | ZNF469 | 84627 | ENSG00000225614 |
| ENSG00000205336 | 1986,145 | -1,01123 | 0,44537 | 0,00088 | 0,040909 | ADGRG1 | 9289 | ENSG00000205336 |
| ENSG00000115523 | 3107,328 | -1,01364 | 0,335156 | 0,000114 | 0,009238 | GNLY | 10578 | ENSG00000115523 |
| ENSG00000275199 | 401,2848 | -1,02775 | 0,30444 | 3,57E-05 | 0,003681 | AKT3 | 10000 | ENSG00000275199 |
| ENSG00000205189 | 229,7861 | -1,03245 | 0,279803 | 1,15E-05 | 0,001611 | ZBTB10 | 65986 | ENSG00000205189 |
| ENSG00000134539 | 469,4947 | -1,04968 | 0,286026 | 1,22E-05 | 0,001685 | KLRD1 | 3824 | ENSG00000134539 |
| ENSG00000185697 | 675,0417 | -1,05259 | 0,274601 | 6,47E-06 | 0,001005 | MYBL1 | 4603 | ENSG00000185697 |
| ENSG00000137441 | 110,4538 | -1,06568 | 0,48637 | 0,000992 | 0,044449 | FGFBP2 | 83888 | ENSG00000137441 |
| ENSG00000030419 | 333,174 | -1,07393 | 0,438838 | 0,00055 | 0,030158 | IKZF2 | 22807 | ENSG00000030419 |
| ENSG00000150045 | 261,5656 | -1,11596 | 0,432912 | 0,000384 | 0,023056 | KLRF1 | 51348 | ENSG00000150045 |
| ENSG00000180739 | 152,3445 | -1,14498 | 0,451298 | 0,000421 | 0,024811 | S1PR5 | 53637 | ENSG00000180739 |
| ENSG00000100453 | 794,5134 | -1,16052 | 0,289261 | 2,99E-06 | 0,000531 | GZMB | 3002 | ENSG00000100453 |
| ENSG00000134545 | 80,59457 | -1,18043 | 0,48183 | 0,000527 | 0,029346 | KLRC1 | 3821 | ENSG00000134545 |
| ENSG00000019485 | 78,1 | -1,18735 | 0,422353 | 0,000198 | 0,01365 | PRDM11 | 56981 | ENSG00000019485 |
| ENSG00000116667 | 104,4928 | -1,189 | 0,440273 | 0,000268 | 0,017454 | C1orf21 | 81563 | ENSG00000116667 |
| ENSG00000146070 | 232,4877 | -1,19905 | 0,356346 | 3,35E-05 | 0,003584 | PLA2G7 | 7941 | ENSG00000146070 |
| ENSG00000237651 | 58,71064 | -1,20055 | 0,466782 | 0,000385 | 0,023056 | C2orf74 | 339804 | ENSG00000237651 |
| ENSG00000007264 | 521,5335 | -1,2034 | 0,260029 | 1,88E-07 | 5,91E-05 | MATK | 4145 | ENSG00000007264 |
| ENSG00000157985 | 205,8823 | -1,2295 | 0,311862 | 3,83E-06 | 0,000657 | AGAP1 | 116987 | ENSG00000157985 |
| ENSG00000006634 | 142,2305 | -1,23993 | 0,436405 | 0,000175 | 0,012381 | DBF4 | 10926 | ENSG00000006634 |
| ENSG00000007402 | 201,7487 | -1,24381 | 0,508945 | 0,000504 | 0,02823 | CACNA2D2 | 9254 | ENSG00000007402 |
| ENSG00000232653 | 1656,379 | -1,24518 | 0,554365 | 0,000777 | 0,037699 | GOLGA8N | 643699 | ENSG00000232653 |
| ENSG00000149294 | 219,8555 | -1,25162 | 0,336695 | 9,15E-06 | 0,001349 | NCAM1 | 4684 | ENSG00000149294 |
| ENSG00000232126 | 31320,4 | -1,26439 | 0,448235 | 0,000181 | 0,012762 | HLA-B | 3106 | ENSG00000232126 |
| ENSG00000102543 | 143,9574 | -1,27292 | 0,357848 | 1,62E-05 | 0,002044 | CDADC1 | 81602 | ENSG00000102543 |
| ENSG00000211689 | 552,0221 | -1,28347 | 0,589916 | 0,000892 | 0,04121 | TRGC1 | 6966 | ENSG00000211689 |
| ENSG00000174600 | 154,3907 | -1,30465 | 0,559961 | 0,000631 | 0,032742 | CMKLR1 | 1240 | ENSG00000174600 |
| ENSG00000073861 | 446,7611 | -1,31196 | 0,395874 | 3,80E-05 | 0,003864 | TBX21 | 30009 | ENSG00000073861 |
| ENSG00000139116 | 417,1898 | -1,32933 | 0,427888 | 7,53E-05 | 0,00669 | KIF21A | 55605 | ENSG00000139116 |
| ENSG00000105374 | 702,2485 | -1,3439 | 0,262936 | 1,60E-08 | 8,41E-06 | NKG7 | 4818 | ENSG00000105374 |
| ENSG00000211696 | 115,9198 | -1,36138 | 0,451204 | 0,000101 | 0,008419 | TRGV8 | 6982 | ENSG00000211696 |
| ENSG00000166428 | 122,0059 | -1,37794 | 0,560632 | 0,000462 | 0,026214 | PLD4 | 122618 | ENSG00000166428 |
| ENSG00000278362 | 162,2967 | -1,39587 | 0,404544 | 2,36E-05 | 0,002882 | NCR1 | 9437 | ENSG00000278362 |
| ENSG00000158050 | 243,9832 | -1,42548 | 0,701955 | 0,001139 | 0,047557 | DUSP2 | 1844 | ENSG00000158050 |
| ENSG00000150687 | 129,3958 | -1,43569 | 0,418803 | 2,47E-05 | 0,002982 | PRSS23 | 11098 | ENSG00000150687 |
| ENSG00000151612 | 188,6611 | -1,44573 | 0,446633 | 4,79E-05 | 0,004649 | ZNF827 | 152485 | ENSG00000151612 |
| ENSG00000196867 | 86,59228 | -1,54004 | 0,652054 | 0,000541 | 0,0299 | ZFP28 | 140612 | ENSG00000196867 |
| ENSG00000173068 | 128,2675 | -1,67052 | 0,767414 | 0,000783 | 0,037838 | BNC2 | 54796 | ENSG00000173068 |
| ENSG00000120156 | 46,99189 | -1,68022 | 0,7273 | 0,0006 | 0,031723 | TEK | 7010 | ENSG00000120156 |
| ENSG00000069702 | 2591,686 | -1,81015 | 0,30174 | 9,34E-11 | 7,65E-08 | TGFBR3 | 7049 | ENSG00000069702 |
| ENSG00000100450 | 615,0176 | -1,81829 | 0,460781 | 3,20E-06 | 0,000561 | GZMH | 2999 | ENSG00000100450 |
| ENSG00000211695 | 74,19579 | -1,95397 | 0,726289 | 0,000219 | 0,014817 | TRGV9 | 6983 | ENSG00000211695 |
| ENSG00000205809 | 169,4629 | -1,98721 | 0,70239 | 0,000147 | 0,011075 | KLRC2 | 3822 | ENSG00000205809 |
| ENSG00000281123 | 873,2644 | -2,08546 | 0,597168 | 1,71E-05 | 0,002138 | FCGBP | 8857 | ENSG00000281123 |
| ENSG00000274320 | 595,659 | -2,11005 | 0,741859 | 0,000134 | 0,010327 | GOLGA6L9 | 440295 | ENSG00000274320 |
| ENSG00000113070 | 62,68808 | -2,2564 | 0,810349 | 0,000169 | 0,012084 | HBEGF | 1839 | ENSG00000113070 |
| ENSG00000176083 | 84,91998 | -2,29672 | 0,697563 | 3,45E-05 | 0,003633 | ZNF683 | 257101 | ENSG00000176083 |
| ENSG00000089169 | 72,36408 | -2,31479 | 0,836921 | 0,000149 | 0,011127 | RPH3A | 22895 | ENSG00000089169 |
| ENSG00000117595 | 28,59907 | -2,31915 | 0,81995 | 0,000119 | 0,009557 | IRF6 | 3664 | ENSG00000117595 |
| ENSG00000183542 | 228,5933 | -2,37092 | 0,548146 | 6,16E-07 | 0,000147 | KLRC4 | 8302 | ENSG00000183542 |
| ENSG00000107249 | 23,42067 | -2,45309 | 0,743083 | 3,22E-05 | 0,003487 | GLIS3 | 169792 | ENSG00000107249 |
| ENSG00000142178 | 81,95144 | -2,63933 | 0,811214 | 3,52E-05 | 0,003658 | SIK1 | 150094 | ENSG00000142178 |
| ENSG00000078114 | 223,4598 | -4,94084 | 0,666798 | 5,85E-16 | 2,16E-12 | NEBL | 10529 | ENSG00000078114 |

**Table S3:** Purity of isolated NK-cells in % of CD3^-^, CD56^+^ cells.

| **Donor** | **condition** | **purity of isolated NK cells ( % CD3-, CD56+)** |
| --- | --- | --- |
| K16 | healthy | 90,1 |
| K17 | healthy | 82,8 |
| K18 | healthy | 90,1 |
| K19 | healthy | 92,5 |
| K21 | healthy | 86,8 |
| K24 | healthy | 83,8 |
| K27 | healthy | 85,6 |
| K30 | healthy | 94,5 |
| K31 | healthy | 92,2 |
| K33 | healthy | 85,8 |
| K34 | healthy | 82,5 |
| K6 | healthy | 94,7 |
| 83 | renal | 89 |
| 102 | renal | 90,7 |
| 110 | renal | 90,3 |
| 117 | renal | 87,2 |
| 122 | renal | 92,1 |
| 124 | renal | 80,4 |
| 138 | renal | 92,2 |
| 139 | renal | 94,7 |
| 145 | renal | 86,5 |
| 151 | renal | 82,5 |
| 101 | skin | 92,2 |
| 105 | skin | 94,6 |
| 108 | skin | 95,8 |
| 121 | skin | 72,7 |
| 123 | skin | 95,8 |
| 126 | skin | 90,4 |
| 150 | skin | 82,1 |
